# GENERAL PRINCIPLES OF SINGLE-CONSTRUCT CHROMOSOMAL GENE DRIVE

**DOI:** 10.1111/j.1558-5646.2012.01582.x

**Published:** 2012-07

**Authors:** John M Marshall, Bruce A Hay

**Affiliations:** 1 Division of Biology, California Institute of TechnologyPasadena, CA 91125; 3 MRC Centre for Outbreak Analysis and Modelling, Department of Infectious Disease Epidemiology, School of Public Health, Imperial College LondonLondon W2 1PG, United Kingdom

**Keywords:** Antidote, lepidopteron pests, population replacement, population suppression, toxin, transgenic mosquitoes

## Abstract

Gene drive systems are genetic elements capable of spreading into a population even if they confer a fitness cost to their host. We consider a class of drive systems consisting of a chromosomally located, linked cluster of genes, the presence of which renders specific classes of offspring arising from specific parental crosses unviable. Under permissive conditions, a number of these elements are capable of distorting the offspring ratio in their favor. We use a population genetic framework to derive conditions under which these elements spread to fixation in a population or induce a population crash. Many of these systems can be engineered using combinations of toxin and antidote genes, analogous to *Medea*, which consists of a maternal toxin and zygotic antidote. The majority of toxin–antidote drive systems require a critical frequency to be exceeded before they spread into a population. Of particular interest, a Z-linked *Medea* construct with a recessive antidote is expected to induce an all-male population crash for release frequencies above 50%. We suggest molecular tools that may be used to build these systems, and discuss their relevance to the control of a variety of insect pest species, including mosquito vectors of diseases such as malaria and dengue fever.

Gene drive occurs when genetic elements—including genes, gene complexes, entire chromosomes, and endosymbiotic bacteria—increase in frequency in a population even if their presence results in a fitness cost. Gene drive has long been proposed as a mechanism for controlling insect pest populations, beginning with the suggestion by Serebrovskii (1940) that chromosomal translocations be driven into pest populations, thereby inducing a fitness load and suppressing population size. Curtis (1968) pointed out that this same method could be used to drive disease-refractory genes into mosquito populations. Early control strategies relied on irradiation to induce chromosomal abnormalities and were of limited success (reviewed in Gould and Schliekelman 2004); however, recent advances in molecular biology and insect transgenesis provide new methods for achieving these goals. Gene drive is now being considered to promote the spread of a variety of desirable traits into pest populations—notably the inability of insects to transmit diseases to humans, animals, and plants (Braig and Yan 2001; Sinkins and Gould 2006). Novel approaches are also being considered to suppress pest populations by spreading genes that induce a fitness cost, or that bias the offspring gender ratio, eventually leading to a single-gender population crash (Burt 2003).

A variety of gene drive systems have now been characterized (Sinkins and Gould 2006), and different systems are appropriate depending on their intended range of use. If the goal is to spread transgenes over large geographical areas, then invasive gene drive systems, which are capable of spreading from one population to another, even in the presence of low migration rates, are most appropriate. Several gene drive systems display this property, many of which have been observed to spread in wild populations. Examples include transposable elements (Engels 1989), male meiotic drive systems (Hickey and Craig 1966), homing endonuclease genes (HEGs) (Gimble 2000), *Medea* elements (Beeman et al. 1992), and the endosymbiont *Wolbachia* (Turelli and Hoffmann 1991). Each of these naturally occurring elements presumably spread from a single site, suggesting that synthetic elements engineered along similar lines (e.g., Windbichler et al. 2011) will also be able to spread geographically beginning from low initial frequencies.

In other circumstances, gene drive systems may be preferred that, while strong enough to bring about population replacement at an isolated release site, are unable to easily establish themselves in neighboring populations. Such systems would be appropriate during the testing phase of population replacement, to allow the population effects of an introduced transgene to be seen on a relatively small scale prior to a potentially global release. They would also be appropriate when approval for a release of transgenic insects is limited to specific regions (Marshall 2010), or when public acceptance is not widespread (Marshall et al. 2010). Gene drive systems of this type display threshold properties in which, above a critical frequency they spread into a population, and below this frequency they are eliminated. Examples include chromosomal translocations (Curtis 1968), inversions (Robinson 1975), compound chromosomes (Foster et al. 1972), and engineered underdominance (Davis et al. 2001). Chromosomal alterations are known to have spread to fixation in many species when considered on an evolutionary timescale (Bush et al. 1977); however, on a human timescale involving many fewer trials, these systems are predicted to remain confined to an isolated release site (Altrock et al. 2010; Marshall and Hay 2012a).

Here, we consider a class of gene drive systems consisting of a single chromosomally located linked cluster of genes (henceforth referred to as a construct) that function by rendering specific classes of offspring arising from specific parental crosses unviable. Under permissive conditions, these systems distort the offspring ratio in favor of the transgenic allele, thus resulting in gene drive. *Medea* is the only gene drive system of this class that has been physically realized, and spreads through the action of two tightly linked genes—a maternally expressed toxin gene, the product of which causes all eggs to become unviable; and a zygotically expressed antidote gene, the product of which rescues *Medea*-bearing eggs from the effects of the toxin (Beeman et al. 1992; Chen et al. 2007). The combined action of the maternal toxin and zygotic antidote confers a selective advantage on *Medea*-bearing individuals by causing the death of non-*Medea*-bearing offspring of heterozygous mothers, thus enabling *Medea* to spread into a population from very low initial frequencies (Wade and Beeman 1994; Ward et al. 2010). *Medea* represents only one of thousands of possible ways in which the offspring ratio can be manipulated through the action of a linked cluster of genes. Only a few of these, such as *Semele* (Marshall et al. 2011), *Merea* (Marshall 2011), and inverse *Medea* (Marshall and Hay 2011), have been considered for their potential to promote population replacement or suppression.

We explore the range of possible chromosomally located, single-construct systems using a simple population genetic framework to derive properties that a construct must have in order to spread to fixation in a population—either at both copies of an homologous locus (henceforth referred to as allele fixation), or at one or both copies of the locus per individual (henceforth referred to as gene fixation). The majority of these systems require a significant threshold frequency to be exceeded before they spread into a population, and for these we derive basic properties of the critical frequency that must be exceeded for the construct to spread. We also investigate constructs that induce a population crash—either by causing a deficiency of one gender over the other, or by driving the population toward an unviable genotype. Both autosomal and sex chromosome-linked constructs are considered. While many of the mathematically derived gene drive systems are prohibitively difficult to engineer, a number can be constructed using conceivable toxin–antidote pairs, and are highlighted following the mathematical analysis.

Several gene drive systems are outside the scope of this article, and are described by other authors. HEGs and meiotic drive elements, for example, increase the frequency of a favored allele in the gametes of heterozygous parents, and are well described by Deredec et al. (2008) and Hartl (1970), respectively. These systems can be used either to spread a gene of interest into a population (Pal and Whitten 1974; Burt 2003; Sinkins and Gould 2006), or to induce a population crash (Hamilton 1967; Lyttle 1976; Burt 2003); however, they tend not to display threshold behavior, making it difficult to spatially confine their release. Two-construct systems such as engineered underdominance (Davis et al. 2001) do display threshold behavior, but require a different mathematical treatment than single-construct systems and therefore are beyond the scope of this analysis. That said; a large number of gene drive systems are within the scope of this article, many of which display unique dynamical properties that distinguish them from the gene drive systems currently under consideration. We discuss the relevance of these systems to vector-borne disease control and pest management.

## Model Development

We use a simple difference equation framework to model the spread of a gene drive system through a randomly mating population. In doing so, we assume discrete generations and infinite population size. Within this framework, we consider both autosomal, X- and Y-linked constructs. These models are easily extended to species for which males are the homogametic sex.

### AUTOSOMAL CONSTRUCT

We consider a gene drive construct as a single allele, which we denote by “*T*,” and refer to the absence of the construct at the corresponding locus as “*t*.” Certain offspring of certain parental crosses are rendered unviable by the construct, and in our initial model, viability is independent of offspring gender. The proportion of the *k*th generation that are individuals of genotypes *tt*, *Tt*, and *TT* are denoted by *u_k_*, *v_k_*, and *w_k_*, respectively. By considering all possible mating pairs, the genotypes of embryos in the next generation are described by the ratio 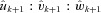, where


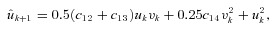
(1)


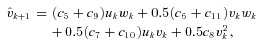
(2)


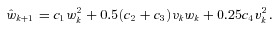
(3)

**Figure 1:**
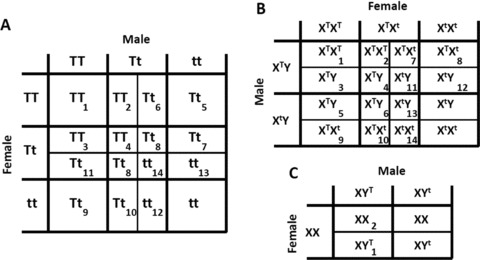
Schematic diagrams representing all possible parental crosses and offspring genotypes for a single-construct gene drive system. A: For the autosomal case, the allele of interest is denoted by “*T*” and the null allele by “*t*,” respectively. We assume that offspring viability is gender-independent. Indices 1–14 represent the 14 ways in which offspring of different parental crosses may be rendered unviable by the construct, and correspond to constants 1 *c*_1_, …, *c*_14_. in Equations 1–3. B: For an X-linked construct, the allele of interest is denoted by “X^*T*^” and the null allele by “X^*t*^.” Indices 1–14 represent the 14 ways in which offspring of different parental crosses may be rendered unviable, and correspond to constants *c*_1_, …, *c*_14_ in Equations 8–12. C: For a Y-linked construct, the allele of interest is denoted by “Y^*T*^” and the null allele by “Y^*t*^.” Here, there are only two ways in which offspring of different parental crosses may be rendered unviable, which correspond to the constants *c*_1_, *c*_2_ in Equations S16–S18.

Here, we have assumed that the viability of heterozygous offspring of two heterozygous parents is independent of which parent donated the *T* allele. Constants *c*_1_, …, *c*_14_ represent the 14 fundamental ways in which offspring of different parental crosses may be rendered unviable by an autosomal construct (Fig. 1A). Each constant is equal to 0 for unviable offspring, and 1 for viable offspring. For the most part, we investigate binary values for these constants; however, for some of our analytical results, incomplete toxicity is included and is denoted by a number between 0 and 1. For example, if a construct renders wild-type offspring of heterozygous mothers and fathers unviable, then *c*_14_= 0 and all other constants are equal to 1. If 10% of affected offspring survive, then *c*_14_= 0.1. For a *Medea* construct, wild-type offspring of heterozygous females and wild-type males are also unviable (Chen et al. 2007) and so *c*_13_, *c*_14_= 0, while all other constants are equal to 1.

With this modeling framework in place, the genotype frequencies in the next generation are given by


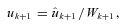
(4)


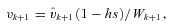
(5)


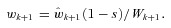
(6)

Here, *s* and *hs* represent the fitness costs associated with being homozygous or heterozygous for the construct, and *W*_*k*+1_ is a normalizing term given by


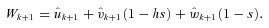
(7)

Using this framework, the dynamic properties of a variety of gene drive systems can be analyzed. In some cases, equilibria can be calculated and their stabilities analyzed analytically. General conditions for a gene drive system to spread to fixation can then be derived. In addition, numerical iterations can determine the general properties of gene drive systems that spread to fixation or induce a population crash, and the threshold behavior that these systems display.

We also consider the dynamics of autosomal gene drive constructs for which offspring are unviable depending on their gender. The modeling framework for this system is cumbersome and is described in the Supporting information.

### X-LINKED CONSTRUCT

Second, we consider the case in which the gene drive construct is inserted at a location on the X chromosome in a species for which males are the heterogametic sex (XY). The dynamics of this system differ due to the fact that females carry two copies of the X chromosome, while males carry one. The proportion of the *k*th generation that are males of genotypes X*^t^*Y and X*^T^*Y are denoted by *u*_*m*,*k*_ and *v*_*m*,*k*_, respectively. Similarly, the proportion that are females of genotypes X*^t^*X*^t^*, X*^T^*X*^t^*, and X*^T^*X*^T^* are denoted by *u*_*f*,*k*_, *v*_*f*,*k*_, and *w*_*f*,*k*_. By considering all possible mating pairs, the genotypes of embryos in the next generation are described by the ratio 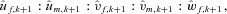 where


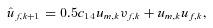
(8)


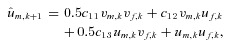
(9)


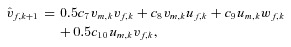
(10)


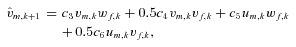
(11)


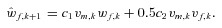
(12)

Here, constants *c*_1_, …, *c*_14_ represent the 14 fundamental ways in which offspring of different parental crosses may be rendered unviable by an X-linked construct (Fig. 1B). Each constant is equal to 0 for unviable offspring and 1 for viable offspring, with intermediate values representing incomplete toxicity. For an X-linked *Medea* construct, wild-type offspring of X*^T^*X*^t^* females are unviable and so *c*_11_, *c*_13_, *c*_14_= 0 and all other constants are equal to 1.

With this modeling framework in place, the genotype frequencies in the next generation are given by


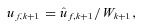
(13)


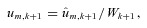
(14)


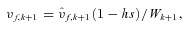
(15)


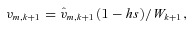
(16)


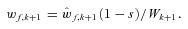
(17)

Here, *s* and *hs* represent the fitness costs associated with being homozygous or heterozygous for the construct, and *W*_*k*+1_ is a normalizing term given by


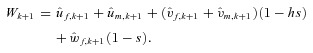
(18)

As for the autosomal case, using this framework, a variety of gene drive systems can be analyzed. General conditions for X-linked gene drive systems to spread to fixation can be derived, and numerical iterations can determine the general properties of gene drive systems that spread to fixation or induce a population crash.

### Y-LINKED CONSTRUCT

Finally, we consider the case in which the construct is inserted at a location on the Y chromosome. The ways in which offspring of different parental crosses may be rendered unviable are shown in Fig 1C and the modeling framework is described in the Supporting information. Numerical iterations of eqs. S16–S18 suggest that loss of the Y-linked toxin–antidote construct is the only stable state whenever the construct has a fitness cost, no matter how small. Y-linked toxin–antidote constructs are therefore not particularly interesting from either an evolutionary or applied perspective.

## Results

We begin by deriving some general requirements for a single-construct system to spread into a population. We then use numerical simulations to validate these requirements, resolve inconclusive cases, and determine which systems induce a population crash.

### AUTOSOMAL CONSTRUCT

Equations 1–7 describe the population frequency of an autosomal construct from one generation to the next. By setting genotype frequencies equal across generations, (*u_k_*, *w_k_*) = (*u*_*k*+1_, *w*_*k*+1_) = (u_*_, w_*_), these equations can be used to calculate stable and unstable equilibria that summarize the dynamics of these constructs. Analytic expressions for these equilibria are too complex to be useful; however, two equilibrium points are expected to exist in most cases—allele fixation, (u_*_, w_*_) = (0, 1), and allele loss, (u_*_, w_*_) = (1, 0). We are interested in the conditions for which allele fixation is locally stable. Under these conditions, the allele of interest will spread to fixation beginning from a range of population frequencies less than 1.

To derive the conditions under which allele fixation is locally stable, we calculate the eigenvalues of the Jacobian matrix,


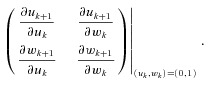
(19)

Allele fixation is locally stable if both eigenvalues have modulus less than 1, and is unstable if one or more of the eigenvalues have modulus greater than 1. If the eigenvalue with the largest modulus has a modulus equal to 1, then the linear stability analysis is inconclusive (Elaydi 1995). We use numerical simulations to resolve inconclusive cases.

Using eqs. 1–7 and 19, we find that allele fixation is associated with eigenvalues equal to 0 and


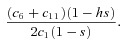
(20)

The second eigenvalue is infinite when *c*_1_= 0 or *s*= 1. This corresponds to the case whereby crosses between *TT* males and *TT* females produce no viable offspring, or when all *TT* individuals are unviable. Allele fixation is not an equilibrium solution to eqs. 1–7 under these conditions, and so it follows that at least partial viability of offspring of *TT* males and *TT* females (*c*_1_ > 0) is a requirement for allele fixation.

The second eigenvalue is equal to 0 when *c*_6_, *c*_11_= 0 or *hs*= 1. This suggests that allele fixation is locally stable when crosses between *TT* and *Tt* individuals produce no viable *Tt* offspring, or when all *Tt* individuals are unviable (i.e., for the case of a completely underdominant allele). If only one of the crosses between *TT* and *Tt* individuals produces unviable *Tt* offspring (e.g., the cross between *TT* males and *Tt* females, in which case *c*_6_= 0 and *c*_11_= 1), then allele fixation is locally stable provided that *s* < 1/(2 −*h*). This condition is satisfied for fitness costs of *s* < 0.5 regardless of heterozygosity (*h*∈ [0, 1]). It is unlikely that gene drive constructs having fitness costs of *s*≥ 0.5 would be deemed suitable for further development, suggesting that any realistic construct will spread to allele fixation above a certain critical population frequency provided that crosses either between *TT* males and *Tt* females or between *Tt* males and *TT* females produce no viable *Tt* offspring.

If all three of the cross outcomes highlighted above are viable (in which case *c*_1_, *c*_6_, *c*_11_= 1), while the viability of other cross outcomes is unspecified, then allele fixation is unstable for nonzero fitness costs (*s* > 0) that are greater in homozygotes than heterozygotes (*h* < 1). However, linear stability analysis is inconclusive in the absence of fitness costs (*s*= 0) or when fitness costs are dominant (*h*= 1). In general, a toxin–antidote construct is predicted to spread to allele fixation when


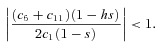
(21)

Here, *c*_1_ represents the proportion of offspring of crosses between *TT* males and *TT* females that are viable; *c*_6_ represents the proportion of *Tt* offspring of crosses between *Tt* males and *TT* females that are viable; and *c*_11_ represents the proportion of *Tt* offspring of crosses between *TT* males and *Tt* females that are viable.

**Figure 2:**
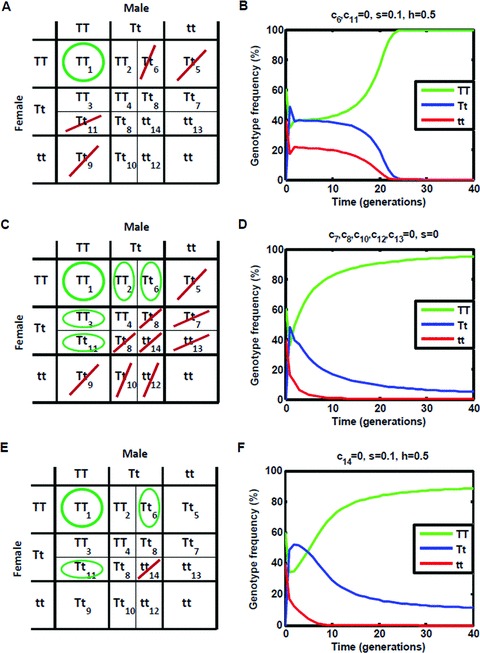
Conditions for the spread of an autosomal gene drive construct. A: Strong condition for allele fixation. An autosomal construct is expected to spread to allele fixation, even in the presence of realistic fitness costs, if *Tt* offspring of crosses between *TT* and *Tt* individuals are rendered unviable (*c*_6_= 0 and/or *c*_11_= 0, highlighted here by red lines), or if all offspring of crosses between *TT* and *tt* individuals are unviable (*c*_5_, *c*_9_= 0, again highlighted by red lines). Another requirement is that crosses between *TT* individuals produce at least partially viable offspring (*c*_1_ > 0, highlighted here by a green circle). B: An example of a construct that satisfies this condition is shown (*c*_6_, *c*_11_= 0). C: Weak condition for allele fixation. An autosomal construct that does not satisfy the strong condition for allele fixation can still spread to allele fixation, in the absence of a fitness cost, if *TT* offspring of crosses between *TT* and *Tt* individuals are viable (*c*_2_, *c*_3_= 1, highlighted here by green ovals), and if *Tt* or *tt* offspring are rendered unviable in at least one parental cross (one of *c*_5_, …, *c*_14_ is equal to 0, highlighted here by red lines, with green ovals for *c*_6_, *c*_11_ to ensure that the strong condition for allele fixation is not satisfied). Crosses between *TT* individuals must also produce at least partially viable offspring (*c*_1_ > 0, green circle). D: An example of a construct that satisfies this condition is shown (*c*_7_, *c*_8_, *c*_10_, *c*_12_, *c*_13_= 0). E: Strong condition for gene fixation. An autosomal construct is expected to spread to gene fixation (fixation of *TT* and *Tt* individuals), even in the presence of realistic fitness costs, if *tt* offspring of crosses between *Tt* individuals are unviable (*c*_14_= 0, red line). The only exception is if all *TT* offspring of parental crosses involving *Tt* parents are rendered unviable (*c*_2_, *c*_3_, *c*_4_= 0), in which case gene fixation will occur if *tt* offspring of crosses between *Tt* and *tt* parents are unviable (*c*_12_= 0 and/or *c*_13_= 0 ). Additionally, crosses between *TT* individuals must produce at least partially viable offspring (*c*_1_ > 0, green circle) and we use green ovals for *c*_6_, *c*_11_ to ensure that the strong condition for allele fixation is not satisfied. F: An example of a construct that satisfies this condition is shown (*c*_14_= 0 ).

Numerical iterations of eqs. 1–7 for the 2^14^− 1 possible combinations of viable and unviable offspring genotypes arising from specific parental crosses (which may each be thought of as the effects of a unique genetic construct) confirm the validity of the above analysis. The simulations also highlight an additional condition for allele fixation—a genetic construct will spread to allele fixation provided that it renders all offspring of parental crosses between *TT* and *tt* individuals unviable (*c*_5_, *c*_9_= 0). This condition holds true under all realistic parameterizations (*h*∈ [0, 1] and *s* < 0.5). The requirements outlined in the above four paragraphs encapsulate what we shall refer to as the strong condition for allele fixation—in which allele fixation occurs despite a nondominant fitness cost—and are visualized in Fig 2A. An example of a construct that satisfies this condition is shown in Fig 2B (*c*_6_, *c*_11_= 0, *h*= 0.5, and *s*= 0.1). It should be noted that constructs outlined in this section are illustrative, and not necessarily straightforward to construct.

Numerical iterations also resolve the inconclusive cases mentioned above. If all three of the highlighted cross outcomes are viable (*c*_1_, *c*_6_, *c*_11_= 1), one or both of the crosses between *TT* and *tt* individuals produce viable offspring (*c*_5_= 1 and/or *c*_9_= 1), and the fitness cost is zero (*s*= 0) or dominant (*h*= 1), then simulations are required to determine the stability of allele fixation. Under these conditions, allele fixation is locally stable provided that *TT* offspring of parental crosses between *TT* and *Tt* individuals are viable (*c*_2_, *c*_3_= 1), and *Tt* or *tt* offspring are rendered unviable in at least one parental cross (one of *c*_5_, …, *c*_14_ is equal to 0). These requirements lead to what we shall refer to as the weak condition for allele fixation—in which allele fixation only occurs if the fitness cost is zero or dominant—and are visualized in Fig 2C. An example of a construct that satisfies this condition is shown in Fig 2D (*c*_7_, *c*_8_, *c*_10_, *c*_12_, *c*_13_= 0 and *s*= 0). Although allele fixation does not occur for these constructs in the presence of a nondominant fitness cost, the constructs can still spread to a high population frequency. For example, if the construct shown in Fig 2D has an additive fitness cost of *s*= 0.1 (*h*= 0.5), it is predicted to reach an allele frequency of 94.7% at equilibrium, with 99.7% of individuals having at least one copy of the allele.

An alternative to allele fixation is gene fixation, in which *TT* and *Tt* individuals fix in the population; but the *T* allele itself does not necessarily fix. This is relevant for transgenic constructs containing dominant effector genes; for example, the disease-refractory genes currently being investigated in mosquitoes all work through dominant mechanisms (Ito et al. 2002; Franz et al. 2006; Corby-Harris et al. 2010). Numerical simulations suggest that, if the conditions for allele fixation are not satisfied, then gene fixation will occur in most cases if *tt* offspring of parental crosses between *Tt* males and *Tt* females are unviable (*c*_14_= 0). The only exception is if all *TT* offspring of parental crosses involving *Tt* individuals are rendered unviable by the construct (*c*_2_, *c*_3_, *c*_4_= 0). In this case, there is an additional requirement for gene fixation to occur—either *tt* offspring of one or both crosses between *Tt* and *tt* parents must be unviable (*c*_12_= 0 and/or *c*_13_= 0), or the fitness cost due to the construct must be zero (*s*= 0) or dominant (*h*= 1). These requirements lead to what we shall refer to as the strong condition for gene fixation—in which gene fixation occurs despite a nondominant fitness cost—and are visualized in Fig 2E. An example of a construct that satisfies this condition is shown in Fig 2F (*c*_14_= 0, *h*= 0.5, and *s*= 0.1).

Up to this point, we have been considering cases in which crosses between *TT* males and *TT* females produce viable offspring (*c*_1_= 1); however, the dynamics of constructs for which these offspring are unviable (*c*_1_= 0) are also of interest, particularly for applied purposes. Allele fixation is not possible in these cases; however, gene fixation is, and some examples of this are provided in Figure S3. Driving a population toward a genotype that produces no viable offspring is also predicted to induce a population crash in some cases. Several examples are provided in Figure S4; however, none of these appear easy to engineer.

The conditions under which allele fixation is locally stable for an autosomal construct conferring gender-dependent offspring viability are described in the Supporting information. Summarizing these results, an autosomal construct is expected to spread to allele fixation provided that


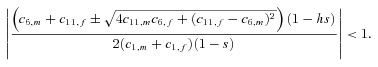
(22)

Here, *c*_1,*m*_ represents the proportion of male offspring of crosses between *TT* males and *TT* females that are viable; *c*_6,*m*_ represents the proportion of male *Tt* offspring of crosses between *Tt* males and *TT* females that are viable; and *c*_11,*m*_ represents the proportion of male *Tt* offspring of crosses between *TT* males and *Tt* females that are viable. The corresponding proportions for female offspring are *c*_1,*f*_, *c*_6,*f*_, and *c*_11,*f*_.

For an autosomal construct that confers female-specific offspring lethality, the condition for allele fixation simplifies to


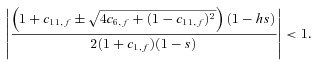
(23)

In order for allele fixation to be an equilibrium solution of eqs. S1–S13, female offspring of crosses between *TT* males and *TT* females must be at least partially viable (*c*_1,*f*_ > 0). This is therefore a requirement for allele fixation. Furthermore, analysis of eq. 23 suggests that, if either cross between *TT* and *Tt* individuals produces unviable female *Tt* offspring (*c*_6,*f*_= 0 and/or *c*_11,*f*_= 0), then allele fixation is locally stable for modest fitness costs. If all of these highlighted cross outcomes produce viable female offspring (*c*_1,*f*_, *c*_6,*f*_, *c*_11,*f*_= 1), and the fitness cost is zero (*s*= 0) or dominant (*h*= 1), then simulations are required to determine the stability of allele fixation. In these cases, allele fixation is locally stable provided that female *TT* offspring of parental crosses between *TT* and *Tt* individuals are viable (*c*_2,*f*_, *c*_3,*f*_= 1) and female *Tt* or *tt* offspring are rendered unviable in at least one parental cross (one of *c*_5,*f*_, …, *c*_14,*f*_ is equal to 0). Conditions required to induce a population crash are discussed in the Supporting information.

In all cases for which allele fixation is locally stable, the allele of interest is predicted to spread to fixation beginning from a range of population frequencies less than one. In a few cases, this range of frequencies can be derived analytically; but, in general, numerical simulations are necessary because the release threshold is represented by a family of points in genotype space. Stability analysis reveals just one of these points, and we are most interested in the point having no heterozygotes, because population replacement programs are likely to involve releases of only *TT* individuals.

Numerical iterations of eqs. 1–7 for a variety of initial conditions, assuming a release of equal numbers of male and female *TT* individuals into a population of *tt* individuals, reveal three circumstances in which the release threshold can be predicted:

1. If the only unviable offspring are wild type and the construct has no fitness cost, then the release threshold is zero. Otherwise, the release threshold is greater than zero.
2. If the only unviable offspring are heterozygous and the construct has no fitness cost, then the release threshold is 50%.
3. If only one parental cross gives rise to unviable offspring and the construct has no fitness cost, then the release threshold is equal to the expected proportion of *T* alleles among the unviable offspring.

The first two rules apply to all autosomal constructs; while the third rule applies to constructs for which offspring lethality is bisex-lethal, male-lethal, or female-lethal. To demonstrate the application of the third rule, imagine a construct that renders female *Tt* and *tt* offspring of crosses between *Tt* males and *Tt* females unviable; but affects no other parental crosses. *Tt* offspring have one *T* allele and one *t* allele and are expected to be twice as numerous as *tt* offspring. The proportion of *T* alleles among expected unviable offspring is therefore 2/6, which corresponds to a release threshold of 33.3% for a construct having no fitness cost. When these rules do not apply, the release threshold can be calculated by numerically iterating eqs. 1–7 for a variety of initial conditions. It should be noted that release thresholds vary with the initial distribution of gender and genotype classes. For some constructs, the release threshold is minimized for a female-biased release (Marshall et al. 2011), while for others, it is minimized for a male-biased release.

### X-LINKED CONSTRUCT

Equations 8–18 describe the population frequency of an X-linked construct across generations. By setting genotype frequencies equal from one generation to the next, these equations can be used to calculate stable and unstable equilibria that summarize the dynamics of X-linked constructs. Two equilibrium points are expected to exist in most cases—allele fixation and allele loss—however, the mathematical description of fixation is surprisingly complicated for an X-linked construct with a fitness cost that is less in X*^T^*Y males than in X*^T^*X*^T^* females. We therefore restrict our attention to X-linked constructs with dominant fitness costs (*h*= 1). This is a reasonable simplification because many toxins, antidotes, and effector genes are expected to be dominant in action (e.g., Ito et al. 2002). In these cases, allele fixation can then be represented by the equilibrium, (*u*_*m*,*_, *u*_*f*,*_, *v*_*m*,*_, *w*_*f*,*_) = (0, 0, 0.5, 0.5).

To derive the conditions under which allele fixation is locally stable, we calculate the eigenvalues of the Jacobian matrix,


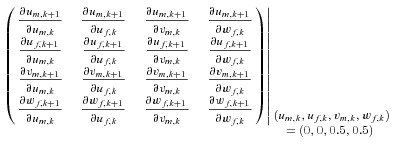
(24)

If all eigenvalues have modulus less than one, then fixation is locally stable (Elaydi 1995), and the allele of interest will spread to fixation beginning from a range of population frequencies less than one.

Using eqs. 8–18 and 24, we find that allele fixation is associated with eigenvalues equal to 0 and


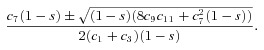
(25)

The second and third eigenvalues are infinite when *c*_1_, *c*_3_= 0 or *s*= 1. This corresponds to the case whereby crosses between X*^T^*Y males and X*^T^*X*^T^* females produce no viable offspring, or when all individuals having the construct are unviable. Allele fixation is not an equilibrium solution to eqs. 8–18 under these conditions, and, in fact, both male and female offspring of this cross must be at least partially viable (*c*_1_, *c*_3_ > 0) in order for the construct to be maintained in the population when fixed. This is therefore a requirement for allele fixation.

**Figure 3:**
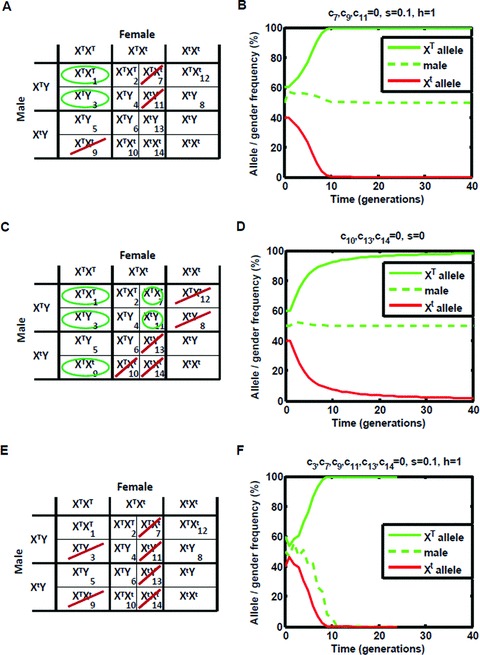
Conditions for an X-linked gene drive construct to spread into a population or induce a population crash. A: Strong condition for allele fixation. An X-linked construct is expected to spread to allele fixation, even in the presence of modest fitness costs, if X^*t*^Y or X^*T*^X^*t*^ offspring of crosses between X^*T*^Y males and X^*T*^X^*t*^ females are rendered unviable (*c*_7_= 0 and/or *c*_11_= 0, red line), or if X^*T*^X^*t*^ offspring of crosses between X^*t*^Y males and X^*T*^X^*T*^ females are unviable (*c*_9_= 0, red line). Another requirement is that crosses between X^*T*^Y males and X^*T*^X^*T*^ females produce at least partially viable female and male offspring (*c*_1_, *c*_3_ > 0, green ovals). B: An example of a construct that satisfies this condition is shown (*c*_7_, *c*_9_, *c*_11_= 0). C: Weak condition for allele fixation. An X-linked construct that does not satisfy the strong condition for allele fixation (*c*_7_, *c*_9_, *c*_11_, = 1, green ovals) can still spread to allele fixation, in the absence of a fitness cost, if the number of *t* alleles among unviable offspring (candidate offspring having one or more *t* allele are crossed with red lines) equals or exceeds the number of *T* alleles among unviable offspring of crosses between X^*T*^Y males and X^*T*^X^*t*^ females and between X^*t*^Y males and X^*T*^X^*T*^ females. The one exception is that offspring of X^*t*^X^*t*^ females are not counted if X^*t*^X^*t*^ offspring are unviable themselves (Equation 27). Crosses between X^*T*^Y males and X^*T*^X^*T*^ females must also produce at least partially viable female and male offspring (*c*_1_, *c*_3_ > 0, green ovals). D: An example of a construct that satisfies this condition is shown (*c*_10_, *c*_13_, *c*_14_, = 0). E: There are several ways in which an X-linked construct can induce a population crash, most of which result in an all female population. The following construct provides one example (*c*_3_, *c*_7_, *c*_9_, *c*_11_, *c*_13_, *c*_14_= 0). F: Beginning from a 60% release proportion, the construct fixes and induces an all-female population crash within 12 generations.

The second and third eigenvalues are equal to 0 when *c*_7_= 0 and either or both of *c*_9_, *c*_11_ are equal to 0. This suggests that allele fixation is locally stable when crosses between X*^T^*Y males and X*^T^*X*^t^* females produce no viable X*^T^*X*^t^* or X*^t^*Y offspring, or alternatively when crosses between X*^T^*Y males and X*^T^*X*^t^* females and crosses between X*^t^*Y males and X*^T^*X*^T^* females produce no viable X*^T^*X*^t^* offspring. Interestingly, if only one of these crosses produces unviable X*^t^*Y or X*^T^*X*^t^* offspring (i.e., if only one of *c*_7_, *c*_9_, *c*_11_ is equal to 0), then allele fixation is still locally stable for all realistic fitness costs (*s* < 0.5). The requirements outlined in the above two paragraphs encapsulate the strong condition for allele fixation of an X-linked construct—in which allele fixation occurs despite a fitness cost—and are visualized in Fig 3A. An example of a construct that satisfies this condition is shown in Fig 3B (*c*_7_, *c*_9_, *c*_11_= 0, *h*= 1, and *s*= 0.1). Once again, constructs outlined in this section are illustrative, and not necessarily straightforward to construct.

If all five of the cross outcomes highlighted are viable (*c*_1_, *c*_3_, *c*_7_, *c*_9_, *c*_11_= 1), while the viability of other cross outcomes is unspecified, then allele fixation is unstable for nonzero fitness costs (*s* > 0). However, linear stability analysis is inconclusive in the absence of a fitness cost (*s*= 0). In general, an X-linked gene drive construct with a dominant fitness cost is predicted to spread to allele fixation when


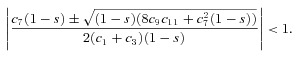
(26)

Here, *c*_1_ and *c*_3_ represent the proportions of X*^T^*X*^T^* and X*^T^*Y offspring of crosses between X*^T^*Y males and X*^T^*X*^T^* females that are viable; *c*_7_ and *c*_11_ represent the proportions of X*^T^*X*^t^* and X*^t^*Y offspring of crosses between X*^T^*Y males and X*^T^*X*^t^* females that are viable; and *c*_9_ represents the proportion of X*^T^*X*^t^* offspring of crosses between X*^t^*Y males and X*^T^*X*^T^* females that are viable.

Numerical iterations of eqs. 8–18 for the 2^14^− 1 possible combinations of viable and unviable offspring genotypes arising from specific parental crosses (each representing a dynamically unique X-linked construct) confirm the validity of the above analysis while resolving the inconclusive cases mentioned above. If all five of the highlighted cross outcomes produce viable offspring (*c*_1_, *c*_3_, *c*_7_, *c*_9_, *c*_11_= 1), and there is no fitness cost (*s*= 0), then simulations are required to determine the stability of allele fixation. Under these conditions, allele fixation is locally stable provided that the following rule is satisfied,


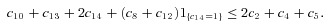
(27)

Here, we have assumed binary values for all constants. The meaning of this equation is that, if the strong condition for allele fixation is not satisfied (i.e., if *c*_1_, *c*_3_, *c*_7_, *c*_9_, *c*_11_= 1) and if there is no fitness cost (*s*= 0), then allele fixation will only occur if the number of *t* alleles among unviable offspring equals or exceeds the number of *T* alleles among unviable offspring of crosses between X*^T^*Y males and X*^T^*X*^t^* females and between X*^t^*Y males and X*^T^*X*^T^* females. The one exception, illustrated by the indicator function, 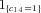, is that offspring of X*^t^*X*^t^* females are not counted if X*^t^*X*^t^* offspring are unviable themselves. This is the weak condition for allele fixation of an X-linked construct—in which allele fixation only occurs in the absence of a fitness cost—and is visualized in Fig 3C. An example of a construct that satisfies this condition is shown in Fig 3D (*c*_10_, *c*_13_, *c*_14_= 0 and *s*= 0).

Thus far, we have been considering cases in which crosses between X*^T^*Y males and X*^T^*X*^T^* females produce viable male and female offspring (*c*_1_, *c*_3_= 1); however, the dynamics of constructs for which this is not the case (*c*_1_= 0 and/or *c*_3_= 0) are also of interest, particularly for applied purposes. Allele fixation is not possible in these cases; however, gene fixation is. Normally, the conditions that lead to gene fixation—unviable X*^t^*X*^t^* offspring produced by crosses between X*^T^*X*^t^* females and X*^t^*Y males (*c*_14_= 0), and unviable X*^t^*Y offspring produced by crosses between X*^T^*X*^t^* females and X*^T^*Y males (*c*_11_= 0)—also lead to allele fixation. However, if crosses between X*^T^*X*^T^* females and X*^T^*Y males also produce unviable female offspring (*c*_1_= 0), then genotypes including the allele of interest fix in the population while the allele itself does not. Some examples of this are provided in Figure S5.

An X-linked construct is also able to induce a population crash if male and/or female offspring of crosses between X*^T^*X*^T^* females and X*^T^*Y males are unviable (*c*_1_= 0 and/or *c*_3_= 0). Numerical iterations for the 2^14^− 1 possible X-linked constructs reveal a number of possibilities. For an all-male population crash, it is required that all female offspring of crosses between X*^T^*Y males and X*^T^*X*^T^* females are unviable (*c*_1_= 0); for an all-female population crash, it is required that all male offspring from this cross must are unviable (*c*_3_= 0); and to drive the population toward an unviable genotype, both genders are required to be unviable (*c*_1_, *c*_3_= 0). One configuration that leads to a population crash (*c*_3_, *c*_7_, *c*_9_, *c*_11_, *c*_13_, *c*_14_= 0) is shown in Fig 3E–F, and several others are provided in Figure S6. Unlike for autosomal constructs, it is conceivable that an X-linked construct that induces a population crash could be engineered using realistic combinations of toxins and antidotes. However, it should be noted that, as population sizes become small, stochastic effects become relevant and a population on the verge of extinction may recover if, by chance, the drive element is eliminated while the wild-type allele is still present (Rigaud et al. 1992; Hatcher et al. 1998).

In all cases for which allele fixation is locally stable, the construct is predicted to spread to fixation beginning from a range of population frequencies less than one. For an X-linked construct, this range is described by a release threshold, which is the minimum population frequency that X*^T^*X*^T^* and X*^T^*Y individuals must be released at in order to spread to fixation. Numerical iterations of eqs. 8–18 for a variety of initial conditions, assuming a release of equal numbers of X*^T^*X*^T^* and X*^T^*Y individuals into a population of X*^t^*X*^t^* and X*^t^*Y individuals, reveal three circumstances, analogous to those for autosomal constructs, in which the release threshold can be predicted:

1. If the only unviable offspring are X*^t^*X*^t^* females and X*^t^*Y males and the construct has no fitness cost, then the release threshold is zero. Otherwise, the release threshold is greater than zero.
2. If the only unviable offspring are X*^T^*X*^t^* females and the construct has no fitness cost, then the release threshold is 50%.
3. If only one parental cross gives rise to unviable offspring and the construct has no fitness cost, then the release threshold is equal to the expected number of X*^T^* alleles divided by the expected number of X*^t^* and X*^T^* alleles among the unviable offspring.

To demonstrate the application of the third rule, imagine a construct that renders X*^T^*X*^t^* and X*^T^*Y offspring of crosses between X*^T^*X*^t^* females and X*^t^*Y males unviable. X*^T^*X*^t^* offspring have one X*^T^* allele and one X*^t^* allele and are expected to be equally as numerous as X*^T^*Y offspring. The expected proportion of X*^T^* alleles among unviable offspring is therefore 2/3, which corresponds to a release threshold of 66.7% for a construct having no fitness cost. In other cases, the release threshold can be calculated by numerically iterating eqs. 8–18 for a variety of initial conditions. As for autosomal constructs, these thresholds vary with the initial distribution of genotype classes.

### TOXIN–ANTIDOTE COMBINATIONS

Given the variety of possible crosses and offspring genotypes, literally thousands of autosomal and X-linked gene drive systems are imaginable. We consider a subset of these systems that could be implemented using combinations of toxins and antidotes. A synthetic *Medea* element has been engineered by linking a dominant maternal toxin to a dominant zygotic antidote (Chen et al. 2007). We extend our analysis to consider dominant, recessive, and heterozygous toxins and antidotes that can be expressed maternally, paternally, or zygotically. We also consider toxins that confer general, male-specific, or female-specific offspring lethality.

**Figure 4:**
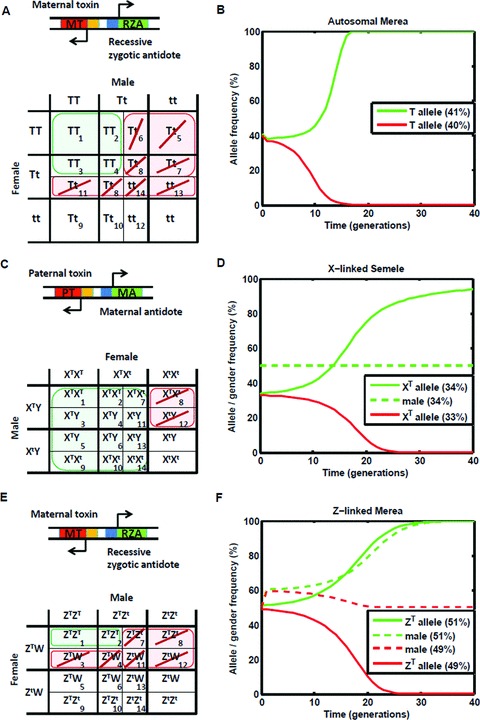
Examples of gene drive systems that can be engineered with simple combinations of toxins and antidotes. A: An autosomal *Medea* construct with a recessive antidote (*c*_5_, *c*_6_, *c*_7_, *c*_8_, *c*_11_, *c*_13_, *c*_14_, = 0). B: This construct satisfies the strong condition for allele fixation and spreads to allele fixation for release proportions greater than 40.8% ( *s*= 0 ). C: An X-linked *Semele* construct (*c*_8_, *c*_12_= 0). D: This construct satisfies the weak condition for allele fixation and, in the absence of a fitness cost ( *s*= 0 ), spreads to allele fixation for release proportions greater than 33.3%. E: A Z-linked *Medea* construct with a recessive antidote. F: This construct induces an all-male population crash for release proportions greater than 50% ( *s*= 0 ).

The general principles derived above can be used to identify a number of gene drive elements. To illustrate their application, let us consider an autosomal *Medea* construct with a recessive antidote (a system we call *Merea*). This construct consists of a dominant maternal toxin linked to a recessive zygotic antidote, represented mathematically as *c*_5_, *c*_6_, *c*_7_, *c*_8_, *c*_11_, *c*_13_, *c*_14_= 0 (Fig. 4A). Here, we see that *c*_1_= 1 and *c*_6_, *c*_11_= 0, satisfying the strong condition for allele fixation and suggesting that the construct will spread to fixation for all realistic fitness costs, provided a sufficiently high release frequency (Fig. 4B).

To provide another example, let us consider an X-linked *Semele* construct consisting of a bisex-lethal paternal toxin and a dominant maternal antidote, represented mathematically as *c*_8_, *c*_12_= 0 (Fig. 4C). Here, we see that the strong condition for allele fixation is not satisfied, but the weak condition is (eq. 27). Only one parental cross is affected, consisting of twice as many X*^t^* alleles as X*^T^* alleles. This suggests that an X-linked *Semele* construct will spread to fixation provided that it has no fitness cost (*s*= 0) and is released at a frequency above 33.3% in a population (Fig. 4D).

Applying these principles and, where necessary, iterating eqs. 1–7 (for autosomal constructs) and eqs. 8–18 (for X-linked constructs), we obtain a list of 53 drive elements that can, in principle, be implemented as toxin–antidote systems that spread to fixation or induce a population crash in the presence of modest fitness costs (Tables 1, S1). For autosomal constructs (Table 1), we see that only systems with recessive antidotes spread to allele fixation in the presence of fitness costs. This is because dominant antidotes rescue *Tt* offspring of crosses between *TT* and *Tt* parents; however, these offspring must be unviable to satisfy the strong condition for allele fixation. Heterozygous toxins also satisfy this condition; however, these may be thought of as dominant toxins linked to recessive antidotes. The condition for gene fixation is satisfied by six toxin–antidote combinations, the simplest being *Medea* and its male equivalent—a dominant paternal toxin linked to a dominant zygotic antidote. No simple autosomal toxin–antidote combinations induce a population crash.

**Table 1:** Autosomal toxin–antidote constructs that fix in a population despite a fitness cost

Toxin	Antidote	Threshold^1^
The following autosomal constructs spread to allele fixation in the presence of modest fitness costs (results apply for general, male-specific, and female-specific offspring lethality):		
Dominant maternal toxin	Recessive paternal antidote	50% (46.5%, 53.5%)
Dominant maternal toxin	Recessive zygotic antidote	40.8% (35.5%, 39.6%)
Recessive maternal toxin	Recessive paternal antidote	63.6% (66.0%, 68.1%)
Recessive maternal toxin	Recessive zygotic antidote	50% (50%, 50%)
Heterozygous maternal toxin		50% (50%, 50%)
Dominant paternal toxin	Recessive maternal antidote	50% (53.5%, 46.5%)
Dominant paternal toxin	Recessive zygotic antidote	40.8% (39.6%, 35.5%)
Recessive paternal toxin	Recessive maternal antidote	63.6% (68.1%, 66.0%)
Recessive paternal toxin	Recessive zygotic antidote	50% (50%, 50%)
Heterozygous paternal toxin		50% (50%, 50%)
Dominant zygotic toxin	Recessive maternal antidote	59.2% (64.5%, 60.4%)
Dominant zygotic toxin	Recessive paternal antidote	59.2% (60.4%, 64.5%)
Heterozygous zygotic toxin		50% (50%, 50%)
The following autosomal constructs spread to allele fixation in the presence of modest fitness costs (results apply for general, male-specific, and female-specific offspring lethality):		
Dominant maternal toxin	Dominant zygotic antidote	0%
Heterozygous maternal toxin	Recessive paternal antidote	38.7%
Heterozygous maternal toxin	Dominant zygotic antidote	0%
Dominant paternal toxin	Dominant zygotic antidote	0%
Heterozygous paternal toxin	Recessive maternal antidote	38.7%
Heterozygous paternal toxin	Dominant zygotic antidote	0%

1 Release thresholds are shown for general offspring lethality, with male-specific and female-specific offspring lethality in brackets, respectively.

For X-linked constructs (Table S1), a wide variety of toxin–antidote combinations lead to allele fixation or a population crash. The strong condition for allele fixation is satisfied by 26 X-linked constructs and, unlike the autosomal case, can be achieved without recessive antidotes. Four of these X-linked constructs consist entirely of dominant components—*Medea*, its male equivalent, and a dominant maternal or zygotic toxin linked to a paternal antidote. These constructs also spread to fixation if offspring lethality is male specific (*Medea* and its male equivalent) or female specific (a dominant maternal or zygotic toxin linked to a paternal antidote). The condition for gene fixation is only satisfied by one X-linked construct—a dominant maternal toxin linked to a heterozygous zygotic antidote.

Another distinction between autosomal and X-linked constructs is that simple toxin–antidote combinations inserted at an X-linked locus can induce a population crash. Table S1 lists seven X-linked constructs having this property, all of which cause a crash by creating an all-female population. The simplest example is a bisex-lethal paternal toxin linked to a recessive zygotic antidote. In the absence of a fitness cost (*s*= 0), this construct causes a population crash for release sizes greater than 50%. The other six constructs all consist of a male-lethal paternal toxin linked to a second toxin and sometimes an antidote. These display different threshold behavior but also induce an all-female population crash.

Results for X-linked constructs apply with minor modifications to species for which females are the heterogametic sex (ZW) and males are homogametic (ZZ). For Z-linked constructs, a paternal toxin or antidote becomes a maternal toxin and antidote, and vice versa. The only other change is that a female-lethal toxin becomes a male-lethal toxin, and vice versa. The most significant result for Z-linked constructs is that *Merea* causes a population crash by creating an all-male population (Fig. 4E). Like its X-linked counterpart, in the absence of a fitness cost (*s*= 0), this construct causes a population crash for release sizes greater than 50% (Fig. 4F). The strong condition for allele fixation is satisfied by 26 Z-linked constructs consisting of toxins and antidotes.

## Discussion

We have considered a variety of single-construct gene drive systems that spread by rendering certain offspring genotypes of certain parental crosses unviable, thus distorting the offspring ratio in their favor under permissive conditions. Literally thousands of constructs having this property are imaginable. We have used simple population genetic models to determine the general properties that single-construct gene drive systems must have in order to drive a gene into a population or induce a population crash. We have also identified a subset of these systems that could, in principle, be engineered using linked pairs of genes encoding a toxin and an antidote.

Autosomal constructs are expected to spread to allele fixation in the presence of realistic fitness costs if *Tt* offspring of crosses between *TT* and *Tt* individuals are rendered unviable. Alternatively, if all offspring of crosses between *TT* and *tt* individuals are unviable, the *T* allele will also fix. For allele fixation to occur, it is also required that both male and female offspring of crosses between *TT* individuals are viable. Another possibility is that genotypes including the allele of interest fix in the population while the allele itself does not. This tends to occur if the conditions for allele fixation are not satisfied and *tt* offspring of crosses between *Tt* individuals are unviable. Several possibilities are available for inducing a population crash; however, none of these can be engineered with simple combinations of toxins and antidotes.

Analogous conditions exist for driving an X-linked construct into a population. The first requirement is that male and female offspring of crosses between X*^T^*Y males and X*^T^*X*^T^* females are viable. X-linked constructs are then expected to spread to allele fixation, even in the presence of modest fitness costs, when X*^t^*Y or X*^T^*X*^t^* offspring of crosses between X*^T^*Y males and X*^T^*X*^t^* females are rendered unviable, or when X*^T^*X*^t^* offspring of crosses between X*^t^*Y males and X*^T^*X*^T^* females are unviable. Several possibilities are available for inducing an all-female population crash; some of which can be engineered with combinations of toxins and antidotes. For species in which females are the heterogametic sex, analogous constructs lead to an all-male population crash.

Insect transgenesis is a relatively new field—the first transgenic malaria vector was engineered a little over 10 years ago (Catteruccia et al. 2000)—and it has only been within the last 5 years that synthetic gene drive systems have been engineered in insects, first in *Drosophila melanogaster* with *Medea* (Chen et al. 2007), and more recently in *Anopheles gambiae* with HEGs (Windbichler et al. 2011). Development of *Medea* in *Aedes aegypti* has proved difficult due to a lack of suitable promoters at present (Hay et al. 2010); however, the molecular logics are straightforward. Recent knowledge generated through transcriptional profiling to identify promoters (Xi et al. 2008) and phenotypic characterization of RNAi-mediated effects on the loss or gain –of function of specific genes are providing new opportunities that are now being explored. This suggests that the development time and costs of engineering *Medea* and other toxin–antidote systems in species of interest, such as insect pests and disease vectors, will dramatically decline in the near future. A recent and encouraging result is the development of a two-construct “double *Medea*” system in *D. melanogaster* (Akbari et al., unpubl. ms.), in which each construct possesses a maternal toxin linked to a zygotic antidote for the maternal toxin on the opposite construct. This is the first synthetic drive system to display clear threshold behavior, providing a proof-of-principle for the development of similar systems in other species.

The gene drive systems listed in Table 2 are encouraging because they could conceivably be engineered given our current knowledge of insect genomics and development. Here, we outline toxin and antidote combinations that are likely to retain stability in the face of evolutionary pressures. The toxin gene is the weak point of any toxin–antidote construct because, if mutation leads to its inactivation, the corresponding antidote-only allele may support the reappearance of nontransgenic individuals (Smith 1998). Toxins that are robust to mutational inactivation are therefore desirable. Candidate toxins include small RNAs and proteins that are dominant in action. MicroRNAs designed to silence specific transcripts provide a good example because the functional unit is small (60–80 base pairs), and each microRNA unit in a transcript functions independently, meaning that the toxin gene may consist of multiple microRNAs, each targeting a different sequence and therefore providing functional redundancy in terms of lethality.

**Table 2:** Toxin–antidote constructs that are both biologically feasible and capable of either spreading to fixation or inducing a population crash despite a fitness cost

Toxin	Antidote	Threshold
Autosomal (allele fixation):		
Dominant maternal toxin	Recessive zygotic antidote	40.8%
Dominant paternal toxin	Recessive maternal antidote	50%
Dominant zygotic toxin	Recessive maternal antidote	59.2%
Heterozygous maternal toxin		50%
Heterozygous paternal toxin		50%
Heterozygous zygotic toxin		50%
Autosomal (gene fixation):		
Dominant maternal toxin	Dominant zygotic antidote	0%
X-linked (allele fixation):		
Dominant maternal toxin	Dominant zygotic antidote	0%
Dominant maternal toxin (female-lethal)	Recessive zygotic antidote	40.0%
Paternal toxin	Recessive maternal antidote	44.2%
Dominant zygotic toxin	Recessive maternal antidote	66.7%
Heterozygous maternal toxin		50%
Heterozygous zygotic toxin (female-lethal)		50%
Z linked (population crash):		
Maternal toxin	Recessive zygotic antidote	50%

Recessive toxins may be generated in several ways, each having its own downfalls. First, it may be possible to discover an endogenous gene which, when disrupted at two homologous loci, leads to lethality—either zygotic lethality, or lethality through a maternal or paternal effect. Such a toxin would be robust to evolutionary pressures because it results from the disruption of an endogenous gene; however, it may be difficult to identify a gene whose only effect when lost is to cause a stage-dependent lethality that can then be rescued through the expression of an antidote at another specific life stage. Second, a toxin gene may be generated that is only active as such when inherited from both parents. However, such a toxin gene would be prone to mutational inactivation because mechanisms for bringing about recessivity without dominant killing are expected to involve multiple components (Hay et al., unpubl. ms.), providing several opportunities for the emergence of antidote-only alleles. Therefore, in general, dominant toxins are preferred.

Dominant maternal, paternal, and zygotic toxins are all feasible to engineer. Maternal toxins must be passed to progeny through the female germline, and could consist of mRNAs that encode toxic proteins, or small RNAs that silence the expression of maternally expressed genes whose products are required for embryogenesis (Chen et al. 2007). Paternal toxins, which must be passed to progeny through the sperm or seminal fluid, are more challenging because both sperm and the volume of the ejaculate are small. In consequence, there are probably a limited number of ways in which a paternal toxin can act. Candidate paternal toxins include a nuclease expressed during spermatogenesis that cleaves zygotic chromatin, but not the DNA of the sperm itself; and some progress has been made on this approach (Windbichler et al. 2008). In addition, accessory gland products have been shown to have effects on female behavior, viability, and immunity, demonstrating that it is possible for somatic components of the male ejaculate to influence female physiology (Sirot et al. 2009). Zygotic toxins could consist of small RNAs that silence the expression of an essential gene, or proteins that disrupt development or cell survival. Sex specificity in toxin function may be achieved in several ways—for maternal and zygotic toxins, it may be possible to use the sex determination pathway; and for paternal toxins in species for which males are the heterogametic gender, it may be possible to target the Y chromosome with a sequence-specific nuclease.

Maternal, paternal, and zygotic antidotes are all feasible to engineer; but they may not provide protection against all toxin varieties. A maternal antidote could encode for a protein that neutralizes or degrades a toxin, or a small RNA that promotes degradation of a toxin transcript prior to translation. Mothers provide a large amount of material to their eggs, and hence a maternal antidote may be capable of inhibiting a maternal or paternal toxin, or a zygotic toxin expressed early in development when the maternal effect is still active. A zygotic antidote could encode for a protein or small RNA, and could inhibit a zygotic or maternal toxin, provided that the toxin does not have its effect until after the antidote is expressed. A zygotic antidote is unlikely to be capable of inhibiting a paternal toxin, such as a nuclease expressed in sperm, because zygotic transcription is not initiated until some time after fertilization. A paternally provided antidote could inhibit a paternally provided toxin because both act in the same cells (resulting in paternal underdominance if the toxin is dominant and the antidote is recessive). However, given the limited quantity of material sperm provide to the egg, it is unlikely that a paternal antidote could inhibit a maternal or zygotic toxin.

A large number of the toxin–antidote constructs listed in Tables 1 and S1 use a recessive antidote. Nature has evolved mechanisms capable of detecting twofold differences in gene expression or chromosome number (Sanchez 2008; Keverne 2009; Zakharova et al. 2009). Our understanding of these mechanisms is incomplete; however, synthetic elements having these properties are being investigated and consist of multiple interacting components (Hay et al. unpubl. ms.). Multiple-component antidotes will be prone to mutational inactivation; however, this is not a concern in terms of their evolutionary stability because toxin-only alleles will be rapidly eliminated from any population in which they emerge.

From the above analysis, we identify a number of toxin–antidote systems that are biologically feasible and capable of spreading to fixation or inducing a population crash even in the presence of a fitness cost (Table 2). All of these systems, other than autosomal and X-linked *Medea*, have release thresholds of between 40.0% and 66.7%. Although high, these are much lower than the release ratios achieved for sterile insect programs, which sometimes exceed 90% sterile to wild insects (Krafsur 1998). The existence of a threshold has three advantages during the testing phase of population replacement, or whenever a confined release is preferred—accidentally released insects are unlikely to persist in the wild if released at subthreshold levels; transgenic insects released at superthreshold frequencies at an isolated release site are expected to spread transgenes locally while remaining at subthreshold levels at nearby locations; and transgenes can be eliminated from the release site by diluting them to subthreshold frequencies through a sustained release of wild-type insects (Altrock et al. 2010; Marshall and Hay 2012). Finally, some sex chromosome-linked systems are predicted to induce a population crash at the release site. The most interesting of these systems is Z-linked *Merea*, which is capable of inducing an all-male population crash for releases proportions greater than 50%.

The gene drive systems discussed here are of great relevance to vector-borne disease control and pest management. Mosquito-borne diseases such as dengue fever, chikungunya, and malaria continue to pose a major health problem through much of the world. Treatments for dengue fever and chikungunya remain elusive, and malaria is proving exceptionally difficult to control in highly endemic areas with insecticide-treated nets, indoor residual spraying, and antimalarial drugs (Griffin et al. 2010; World Health Organization 2010). Consequently, there is interest in supplementing currently available control methods with approaches that use gene drive systems to spread disease-refractory genes (e.g., Ito et al. 2002; Franz et al. 2006; Corby-Harris et al. 2010) into wild mosquito populations, or to bring about population suppression (reviewed in Alphey et al. 2002; Sinkins and Gould 2006; Marshall and Taylor 2009).

Another area of application is agricultural pest management. Agricultural pests such as butterflies, beetles, flies, and moths have the potential to significantly reduce yields of a variety of crops, and there is a constant effort to control pest populations without excessive use of pesticides (Kogan 1998). Many insect pests damage crops directly through feeding, while some act as vectors of plant diseases. Control methods using genetically modified crops, such as Bt cotton, have been effective in enhancing the yields of these crops; but many plants of agricultural importance do not express Bt toxin, and some pests, such as the pink bollworm, are becoming resistant (Bagla 2010). Population replacement with insects unable to transmit diseases, and population suppression using gene drive systems that induce a population crash, provide novel, species-specific approaches to insect pest management that complement existing technologies. The results described in this article, while theoretical, outline a number of novel toxin–antidote constructs that could contribute to these goals.

## Supporting information

The following supporting information is available for this article:

**Figure S1.** Schematic diagram representing all possible parental crosses and offspring genotypes for a single-construct gene drive system with gender-dependent offspring viability.

**Figure S2.** Conditions for the spread of an autosomal gene drive construct with gender-dependent offspring viability.

**Figure S3.** Examples of autosomal constructs for which crosses between *TT* males and *TT* females produce noviable offspring (*c*_1_ = 0) that spread to gene fixation.

**Figure S4.** Examples of autosomal constructs for which crosses between *TT* males and *TT* females produce noviable offspring (*c*_1_ = 0 ) that induce a population crash.

**Figure S5.** Examples of X-linked constructs for which crosses between X*^T^*Y males and X*^T^*X*^T^* females produce unviable male and/or female offspring (*c*_1_ = 0 and/or *c*_3_ = 0) that spread to gene fixation.

**Figure S6.** Examples of X-linked constructs for which crosses between X*^T^*Y males and X*^T^*X*^T^* females produce unviable male and/or female offspring (*c*_1_ = 0 and/or *c*_3_ = 0) that induce a population crash.

**Table S1.** X-linked toxin–antidote constructs that fix in a population despite a fitness cost.
