# Supplementary material for: GENERAL PRINCIPLES OF SINGLE-CONSTRUCT CHROMOSOMAL GENE DRIVE
Source: Evolution. 2012 Jul;66(7):2150–66. doi: 10.1111/j.1558-5646.2012.01582.x (PMC3389707; doi:10.1111/j.1558-5646.2012.01582.x)
Supplement: Supplementary file 1 [file evo0066-2150-SD1.pdf]

Supplemental Material:

I. Autosomal construct with gender-dependent offspring viability
II. Y-linked construct
III. Autosomal constructs that lead to gene fixation or a population crash
IV. X-linked constructs that lead to gene fixation or a population crash
V. X-linked toxin-antidote constructs
VI. References

I. Autosomal construct with gender-dependent offspring viability:

Methods: We also consider the dynamics of autosomal gene drive constructs for which offspring viability is gender-dependent. As before, we consider the construct as a single allele denoted by "*T*" and refer to the null allele as "*t*." The proportion of the kth generation that are males of genotypes *tt*, *Tt* and *TT* are denoted by $u_{m,k}$, $v_{m,k}$ and $w_{m,k}$, respectively. The corresponding proportions for females are $u_{f,k}$, $v_{f,k}$ and $w_{f,k}$. By considering all possible mating pairs, the genotypes of embryos in the next generation are described by the ratio $\hat{u}_{f,k+1} : \hat{u}_{m,k+1} : \hat{v}_{f,k+1} : \hat{v}_{m,k+1} : \hat{w}_{f,k+1} : \hat{w}_{m,k+1}$, where,

$$u_{f,k+1} = c_{12,f} v_{m,k} u_{f,k} + c_{13,f} u_{m,k} v_{f,k} + 0.5 c_{14,f} v_{m,k} v_{f,k} + 2 u_{m,k} u_{f,k} \ , \quad \text{(S1)}$$

$$u_{m,k+1} = c_{12,m} v_{m,k} u_{f,k} + c_{13,m} u_{m,k} v_{f,k} + 0.5 c_{14,m} v_{m,k} v_{f,k} + 2 u_{m,k} u_{f,k} \ , \quad \text{(S2)}$$

$$\begin{aligned} v_{f,k+1} &= 2c_{5,f} u_{m,k} w_{f,k} + c_{6,f} v_{m,k} w_{f,k} + c_{7,f} u_{m,k} v_{f,k} + c_{8,f} v_{m,k} v_{f,k} \\ &+ 2c_{9,f} w_{m,k} u_{f,k} + c_{10,f} v_{m,k} u_{f,k} + c_{11,f} w_{m,k} v_{f,k} \end{aligned} \ , \quad \text{(S3)}$$

$$\begin{aligned} v_{m,k+1} &= 2c_{5,m} u_{m,k} w_{f,k} + c_{6,m} v_{m,k} w_{f,k} + c_{7,m} u_{m,k} v_{f,k} + c_{8,m} v_{m,k} v_{f,k} \\ &+ 2c_{9,m} w_{m,k} u_{f,k} + c_{10,m} v_{m,k} u_{f,k} + c_{11,m} w_{m,k} v_{f,k} \end{aligned} \ , \quad \text{(S4)}$$

$$w_{f,k+1} = 2c_{1,f} w_{m,k} w_{f,k} + c_{2,f} v_{m,k} w_{f,k} + c_{3,f} w_{m,k} v_{f,k} + 0.5 c_{4,f} v_{m,k} v_{f,k} \ , \quad \text{(S5)}$$

$$w_{m,k+1} = 2c_{1,m} w_{m,k} w_{f,k} + c_{2,m} v_{m,k} w_{f,k} + c_{3,m} w_{m,k} v_{f,k} + 0.5 c_{4,m} v_{m,k} v_{f,k} \ . \quad \text{(S6)}$$

Here, we have assumed that the viability of heterozygous offspring of two heterozygous

parents is independent of which parent donated the *T* allele. Constants $c_{1,m},...,c_{14,m}$ represent the 14 fundamental ways in which male offspring of different parental crosses may be rendered unviable by the construct (Figure S1). Constants $c_{1,f},...,c_{14,f}$ represent the analogous quantities for female offspring. Each constant is equal to 0 for unviable offspring, and 1 for viable offspring. For the most part, we investigate binary values for these constants; however, for some of our analytical results, incomplete toxicity is included and is denoted by a number between 0 and 1. For a *Medea* construct in which the maternal toxin only kills female offspring, we would have $c_{13,f}, c_{14,f} = 0$ and all other constants would be equal to 1.

Figure S1:

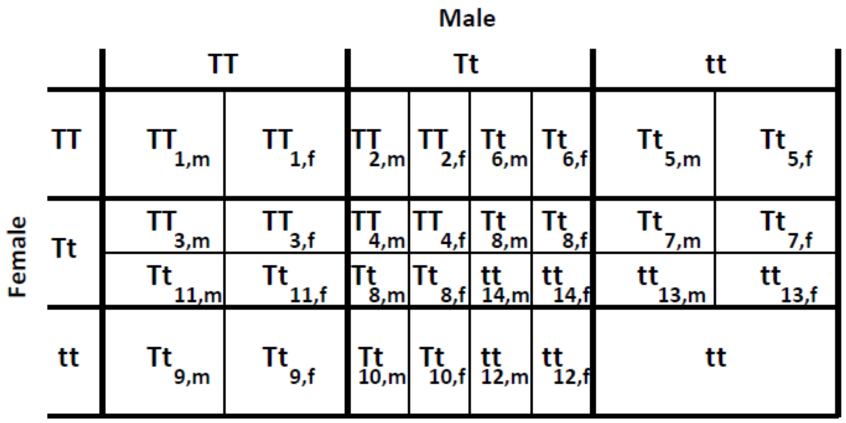

Figure S1. Schematic diagram representing all possible parental crosses and offspring genotypes for a single-construct gene drive system with gender-dependent offspring viability. The allele of interest is denoted by "T" and the null allele by "t," and male and female offspring are denoted by subscripts "m" and "f," respectively. Indices 1-14 represent the 14 ways in which both male and female offspring of different parental crosses may be rendered unviable by the construct, and correspond to constants $c_{1,m},...,c_{14,m}$ and $c_{1,f},...,c_{14,f}$ in Equations S1-S6.

With this modeling framework in place, and assuming equal fitness costs in males and females, the genotype frequencies in the next generation are given by,

$$u_{f,k+1} = \hat{u}_{f,k+1} / W_{k+1} \ , \quad \text{(S7)}$$

$$u_{m,k+1} = \hat{u}_{m,k+1} / W_{k+1} \ , \quad \text{(S8)}$$

$$v_{f,k+1} = \hat{v}_{f,k+1}(1-hs) / W_{k+1} \ , \quad \text{(S9)}$$

$$v_{m,k+1} = \hat{v}_{m,k+1}(1-hs) / W_{k+1} \ , \quad \text{(S10)}$$

$$w_{f,k+1} = \hat{w}_{f,k+1}(1-s) / W_{k+1} \ , \quad \text{(S11)}$$

$$w_{m,k+1} = \hat{w}_{m,k+1}(1-s) / W_{k+1} \ . \quad \text{(S12)}$$

Here, s and hs represent the fitness costs associated with being homozygous or heterozygous for the construct, and W_{k+1} is a normalizing term given by,

$$W_{k+1} = \hat{u}_{f,k+1} + \hat{u}_{m,k+1} + (\hat{v}_{f,k+1} + \hat{v}_{m,k+1})(1-hs) + (\hat{w}_{f,k+1} + \hat{w}_{m,k+1})(1-s) \ . \tag{S13}$$

Using this framework, a variety of gene drive systems for which offspring viability is gender-specific can be analyzed. General conditions for these gene drive systems to spread to fixation can be derived, and numerical iterations can determine the general properties of gene drive systems that spread to fixation or induce a population crash.

Results: Equations S1-S13 describe the population frequency of an autosomal construct with gender-dependent offspring-viability. By setting genotype frequencies equal across generations, these equations can be used to calculate stable and unstable equilibria that summarize the dynamics of these constructs. Two equilibrium points are expected to exist in most cases – allele fixation, $(u_{m,*}, u_{f,*}, v_{m,*}, w_{m,*}, w_{f,*}) = (0,0,0,0.5,0.5)$, and allele loss, $(u_{m,*}, u_{f,*}, v_{m,*}, w_{m,*}, w_{f,*}) = (0.5,0.5,0,0,0)$.

To derive the conditions under which allele fixation is locally stable, we calculate the eigenvalues of the Jacobian matrix,

$$\left.\begin{pmatrix} \frac{\partial u_{m,k+1}}{\partial u_{m,k}} & \frac{\partial u_{m,k+1}}{\partial u_{f,k}} & \frac{\partial u_{m,k+1}}{\partial v_{m,k}} & \frac{\partial u_{m,k+1}}{\partial w_{m,k}} & \frac{\partial u_{m,k+1}}{\partial w_{f,k}} \\ \frac{\partial u_{f,k+1}}{\partial u_{m,k}} & \frac{\partial u_{f,k+1}}{\partial u_{f,k}} & \frac{\partial u_{f,k+1}}{\partial v_{m,k}} & \frac{\partial u_{f,k+1}}{\partial w_{m,k}} & \frac{\partial u_{f,k+1}}{\partial w_{f,k}} \\ \frac{\partial v_{m,k+1}}{\partial u_{m,k}} & \frac{\partial v_{m,k+1}}{\partial u_{f,k}} & \frac{\partial v_{m,k+1}}{\partial v_{m,k}} & \frac{\partial v_{m,k+1}}{\partial w_{m,k}} & \frac{\partial v_{m,k+1}}{\partial w_{f,k}} \\ \frac{\partial w_{m,k+1}}{\partial u_{m,k}} & \frac{\partial w_{m,k+1}}{\partial u_{f,k}} & \frac{\partial w_{m,k+1}}{\partial v_{m,k}} & \frac{\partial w_{m,k+1}}{\partial w_{m,k}} & \frac{\partial w_{m,k+1}}{\partial w_{f,k}} \\ \frac{\partial w_{f,k+1}}{\partial u_{m,k}} & \frac{\partial w_{f,k+1}}{\partial u_{f,k}} & \frac{\partial w_{f,k+1}}{\partial v_{m,k}} & \frac{\partial w_{f,k+1}}{\partial w_{m,k}} & \frac{\partial w_{f,k+1}}{\partial w_{f,k}} \end{pmatrix}\right|_{(u_{m,k}, u_{f,k}, v_{m,k}, w_{m,k}, w_{f,k})=(0,0,0,0.5,0.5)} \ . \tag{S14}$$

If all eigenvalues have modulus less than one, then fixation is locally stable (Elaydi 1995), and the allele of interest will spread to fixation beginning from a range of

population frequencies less than one.

Using Equations S1-S14, we find that allele fixation is associated with eigenvalues equal to 0 and

$$\frac{\left(c_{6,m}+c_{11,f}\pm\sqrt{4c_{11,m}c_{6,f}+(c_{11,f}-c_{6,m})^2}\right)(1-hs)}{2(c_{1,m}+c_{1,f})(1-s)} \quad . \tag{S15}$$

The second and third eigenvalues are infinite when $c_{1,m}, c_{1,f}=0$ or $s=1$. This corresponds to the case whereby crosses between *TT* males and *TT* females produce no viable offspring, or when all *TT* individuals are unviable. Allele fixation is not an equilibrium solution to Equations S1-S13 under these conditions and, in fact, both male and female offspring of this cross must be at least partially viable ($c_{1,m}, c_{1,f}>0$) in order for the construct to be maintained in the population when fixed. This is therefore a requirement for allele fixation.

The second and third eigenvalues are equal to 0 when $c_{6,m}, c_{6,f}, c_{11,m}, c_{11,f}=0$ or $hs=1$. This suggests that allele fixation is locally stable when crosses between *TT* and *Tt* individuals produce no viable *Tt* offspring, or when all *Tt* individuals are unviable (i.e. for the case of a completely underdominant allele), in accordance with conditions for ordinary autosomal constructs. Interestingly, if only one of the crosses between *TT* and *Tt* individuals produces unviable *Tt* offspring, and only male or female offspring are unviable (one of $c_{6,m}, c_{6,f}, c_{11,m}, c_{11,f}$ is equal to 0), then allele fixation is still locally stable for modest fitness costs. If male *Tt* offspring of crosses between *TT* males and *Tt* females are unviable ($c_{11,m}=0$), or if female *Tt* offspring of crosses between *TT* females and *Tt* males are unviable ($c_{6,f}=0$), then fixation is locally stable for all realistic parameterizations ($h\in[0,1]$ and $s<0.5$). However, if female *Tt* offspring of crosses between *TT* males and *Tt* females are unviable ($c_{11,f}=0$), or if male *Tt* offspring of crosses between *TT* females and *Tt* males are unviable ($c_{6,m}=0$), then fixation is locally stable provided that $s<(3-\sqrt{5})/(4-h(1+\sqrt{5}))$. This condition is satisfied under the most

likely parameterizations ($h \in [0,1]$ and $s < 0.19$). Thus, provided that fitness costs are not excessively high, a transgenic construct will spread to allele fixation above a certain release frequency provided that any of $c_{6,m}, c_{6,f}, c_{11,m}, c_{11,f}$ is equal to 0. This requirement encapsulates the strong condition for allele fixation of an autosomal construct with gender-dependent offspring viability – in which allele fixation occurs despite a non-dominant fitness cost – and is visualized in Figure S2A. An example of a construct that satisfies this condition is shown in Figure S2B ($c_{6,f}, c_{11,f}, c_{12,f} = 0$, $h = 0.5$ and $s = 0.1$). It should be noted that constructs outlined in this section are illustrative, and not necessarily straightforward to engineer.

If all three of these highlighted cross outcomes produce viable male and female offspring (in which case $c_{1,m}, c_{1,f}, c_{6,m}, c_{6,f}, c_{11,m}, c_{11,f} = 1$), then allele fixation is unstable for nonzero fitness costs ($s > 0$) that are greater in homozygotes than heterozygotes ($h < 1$) independent of the viability of other cross outcomes. However, linear stability analysis is inconclusive in the absence of a fitness cost ($s = 0$) or when the fitness cost is dominant ($h = 1$). In general, a gene drive construct is predicted to spread to allele fixation when,

$$\left| \frac{\left(c_{6,m} + c_{11,f} \pm \sqrt{4c_{11,m}c_{6,f} + (c_{11,f} - c_{6,m})^2}\right)(1-hs)}{2(c_{1,m} + c_{1,f})(1-s)} \right| < 1 \text{ .} \tag{22}$$

Here, $c_{1,m}$ represents the proportion of male offspring of crosses between *TT* males and *TT* females that are viable; $c_{6,m}$ represents the proportion of male *Tt* offspring of crosses between *Tt* males and *TT* females that are viable; and $c_{11,m}$ represents the proportion of male *Tt* offspring of crosses between *TT* males and *Tt* females that are viable. The corresponding proportions for female offspring are $c_{1,f}$, $c_{6,f}$ and $c_{11,f}$.

For a construct that confers female-specific offspring lethality ($c_{1,m},...,c_{14,m} = 1$), the condition for allele fixation simplifies to,

$$\left|\frac{\left(1+c_{11,f} \pm \sqrt{4c_{6,f}+(1-c_{11,f})^2}\right)(1-hs)}{2(1+c_{1,f})(1-s)}\right|<1 \ . \tag{23}$$

As described earlier, for allele fixation, we require that female offspring of crosses between *TT* males and *TT* females are viable ($c_{1,f}=1$). Also, if either cross between *TT* and *Tt* individuals produces unviable female *Tt* offspring ($c_{6,f}=0$ and/or $c_{11,f}=0$), then allele fixation is locally stable in the presence of modest fitness costs. If all three of these highlighted cross outcomes produce viable female offspring (in which case $c_{1,f},c_{6,f},c_{11,f}=1$), then allele fixation is unstable for nonzero fitness costs ($s>0$) that are greater in homozygotes than heterozygotes ($h<1$). However, linear stability analysis is inconclusive in the absence of a fitness cost ($s=0$) or when the fitness cost is dominant ($h=1$).

Numerical iterations of Equations S1-S13 for the $2^{14}-1$ possible combinations of viable and unviable offspring genotypes arising from specific parental crosses (each representing a unique female-lethal autosomal construct) confirm the validity of the above analysis while resolving the inconclusive cases mentioned above. If all three of the highlighted cross outcomes produce viable female offspring ($c_{1,f},c_{6,f},c_{11,f}=1$), and the fitness cost is zero ($s=0$) or dominant ($h=1$), then simulations are required to determine the stability of allele fixation. Under these conditions, allele fixation is locally stable provided that female *TT* offspring of parental crosses between *TT* and *Tt* individuals are viable ($c_{2,f},c_{3,f}=1$), and female *Tt* or *tt* offspring are rendered unviable in at least one parental cross (one of $c_{5,f},...,c_{14,f}$ is equal to 0). Fixation of *TT* and *Tt* individuals without fixation of the *T* allele is not possible for an autosomal construct conferring female-specific offspring lethality. This is the weak condition for allele fixation of a female-lethal autosomal construct – in which allele fixation only occurs in the absence of a fitness cost – and is visualized in Figure S2C. An example of a construct that satisfies this condition is shown in Figure S2D ($c_{7,f},c_{8,f},c_{10,f},c_{12,f},c_{13,f}=0$ and $s=0$).

Another possibility for an autosomal construct with gender-dependent offspring

viability is to induce a population crash. Earlier, we required that both male and female offspring of crosses between *TT* males and *TT* females be viable ($c_{1,m}, c_{1,f} = 1$) to maintain the *T* allele in the population and hence allow fixation. However, if offspring of one or both genders are unviable ($c_{1,m} = 0$ and/or $c_{1,f} = 0$), the population can be driven towards a population crash. We are most interested in the conditions that lead to an all-male population crash (in which case $c_{1,f} = 0$), since the number of offspring in the next generation is most strongly determined by the number of females. Numerical iterations for the $2^{14} - 1$ possible autosomal constructs conferring female-specific offspring lethality reveal a number of possibilities. All of these require that female offspring of crosses between *TT* and *Tt* individuals are unviable ($c_{6,f}, c_{11,f} = 0$); however, none appear easy to engineer. One configuration that leads to an all-male population crash ($c_{1,f}, c_{2,f}, c_{5,f}, c_{6,f}, c_{8,f}, c_{11,f}, c_{12,f}, c_{13,f}, c_{14,f} = 0$, $h = 0.5$ and $s = 0.1$) is shown in Figure S2E-F.

Figure S2:

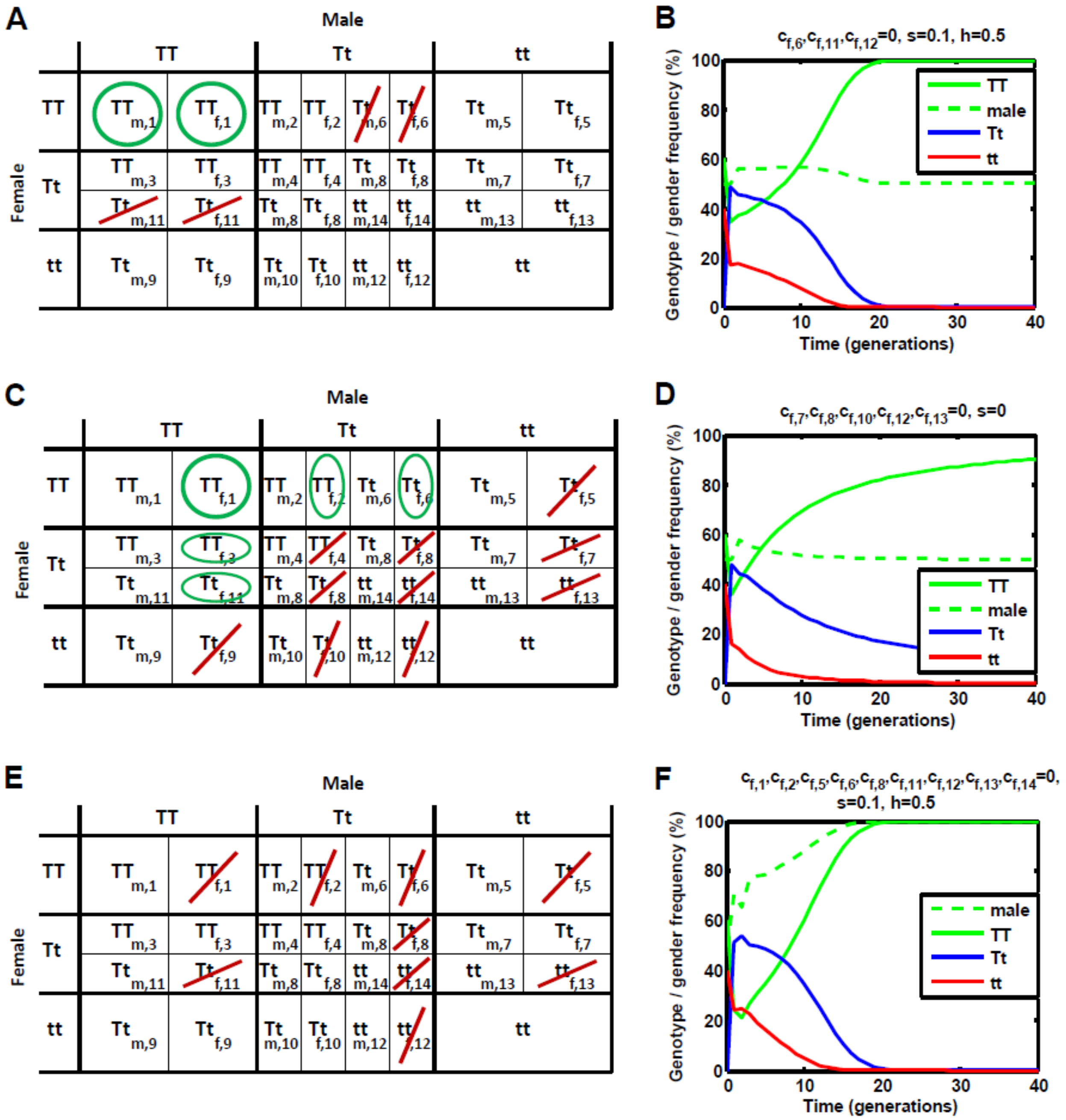

Figure S2. Conditions for the spread of an autosomal gene drive construct with gender-dependent offspring viability. A: Strong condition for allele fixation. An autosomal construct with gender-dependent offspring viability is expected to spread to allele fixation, even in the presence of realistic modest costs, if any male or female *Tt*

offspring of crosses between *TT* and *Tt* individuals are rendered unviable (one of $c_{6,m}, c_{6,f}, c_{11,m}, c_{11,f}$ is equal to 0, red lines). Another requirement is that crosses between *TT* individuals produce at least partially viable male and female offspring ($c_{1,m}, c_{1,f} > 0$, green circles). B: An example of a construct that satisfies this condition is shown ($c_{6,f}, c_{11,f}, c_{12,f} = 0$). C: Weak condition for allele fixation. A female-lethal autosomal construct that does not satisfy the strong condition for allele fixation can still spread to allele fixation, in the absence of a fitness cost, if female *TT* offspring of parental crosses between *TT* and *Tt* individuals are viable ($c_{2,f}, c_{3,f} = 1$, green ovals), and female *Tt* or *tt* offspring are rendered unviable in at least one parental cross (one of $c_{5,f}, ..., c_{14,f}$ is equal to 0, red lines, with green ovals for $c_{6,f}, c_{11,f}$ to ensure that the strong condition for allele fixation is not satisfied). Crosses between *TT* individuals must also produce at least partially viable female offspring ($c_{1,f} > 0$, green circle). D: An example of a construct that satisfies this condition is shown ($c_{7,f}, c_{8,f}, c_{10,f}, c_{12,f}, c_{13,f} = 0$). E: There are several ways in which an autosomal construct with gender-dependent offspring viability can induce a population crash. The following construct provides one example ($c_{1,f}, c_{2,f}, c_{5,f}, c_{6,f}, c_{8,f}, c_{11,f}, c_{12,f}, c_{13,f}, c_{14,f} = 0$). F: Beginning from a 60% release proportion, the construct fixes and induces and all-male population crash within 20 generations.

II. Y-linked construct:

Methods: We consider the case in which the gene drive construct is present at a location on the Y chromosome in a species for which males are the heterogametic sex. The dynamics of this system are much simpler due to the fact that only males carry the Y chromosome. The proportion of the kth generation that are males of genotypes XY^t and XY^T are denoted by $u_{m,k}$ and $v_{m,k}$, respectively; and a proportion, $u_{f,k}$, are females of genotype XX. By considering the two possible mating pairs, the genotype frequencies of embryos in the next generation are given by,

$$u_{m,k+1} = u_{m,k}u_{f,k} / (c_1 v_{m,k} u_{f,k}(1-hs) + c_2 v_{m,k} u_{f,k} + 2u_{m,k}u_{f,k}) \ , \qquad \text{(S16)}$$

$$v_{m,k+1} = c_1 v_{m,k} u_{f,k}(1-hs) / (c_1 v_{m,k} u_{f,k}(1-hs) + c_2 v_{m,k} u_{f,k} + 2u_{m,k}u_{f,k}) \ , \qquad \text{(S17)}$$

$$u_{f,k+1} = (c_2 v_{m,k} u_{f,k} + u_{m,k}u_{f,k}) / (c_1 v_{m,k} u_{f,k}(1-hs) + c_2 v_{m,k} u_{f,k} + 2u_{m,k}u_{f,k}) \ . \qquad \text{(S18)}$$

Here, hs represents the fitness cost associated with the construct, and constants c_1 and c_2 represent the two types of offspring that may be rendered unviable by a Y-linked construct – male and female offspring of XY^T males, respectively (Figure 1C). Each constant is equal to 0 for unviable offspring, and 1 for viable offspring.

Results: Equations S16-S18 describe the population frequency of a Y-linked construct across generations. By setting genotype frequencies equal from one generation to the next, these equations can be used to calculate equilibria that summarize the dynamics of these constructs.

We begin by requiring that XY^T offspring of crosses between XY^T males and XX females are viable ($c_1 = 1$) so that the transgenic construct is maintained in the population under permissive conditions. This restricts our attention to constructs for which XX offspring of crosses between XY^T males and XX females are unviable ($c_2 = 0$). First considering the case of a construct having no fitness cost ($s = 0$), we see by substitution that, if XY^T males are released at an initial frequency of $v_{m,0}$ in the population, they are maintained at this frequency in the population indefinitely. Stability analysis reveals that, in the presence of a fitness cost, loss of the Y^T allele is the only stable equilibrium. These results are confirmed by numerical iteration of Equations S16-S18, and suggest that Y-linked toxin-antidote constructs are not useful for population replacement.

III. Autosomal constructs that lead to gene fixation or a population crash:

Figure S3:

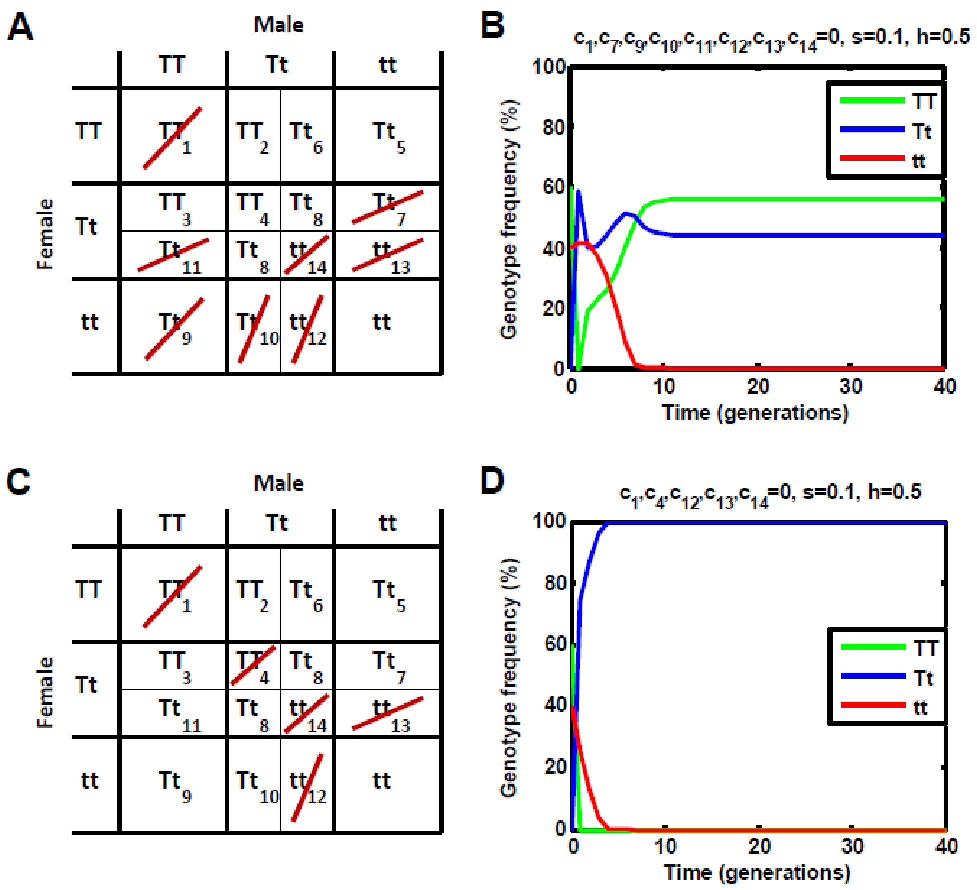

Figure S3. Examples of autosomal constructs for which crosses between *TT* males and *TT* females produce no viable offspring ($c_1 = 0$) that spread to gene fixation. A: The following construct provides one example ($c_1, c_7, c_9, c_{10}, c_{11}, c_{12}, c_{13}, c_{14} = 0$). B: Beginning from a 60% release, wild-type individuals are eliminated within eight generations and the resulting population is a mixture of *Tt* and *TT* individuals. C: The following construct provides another example ($c_1, c_4, c_{12}, c_{13}, c_{14} = 0$). D: Beginning from a 60% release, the population becomes entirely heterozygous within five generations. We reiterate that the constructs outlined in this section are illustrative, and not necessarily straightforward to construct.

Figure S4:

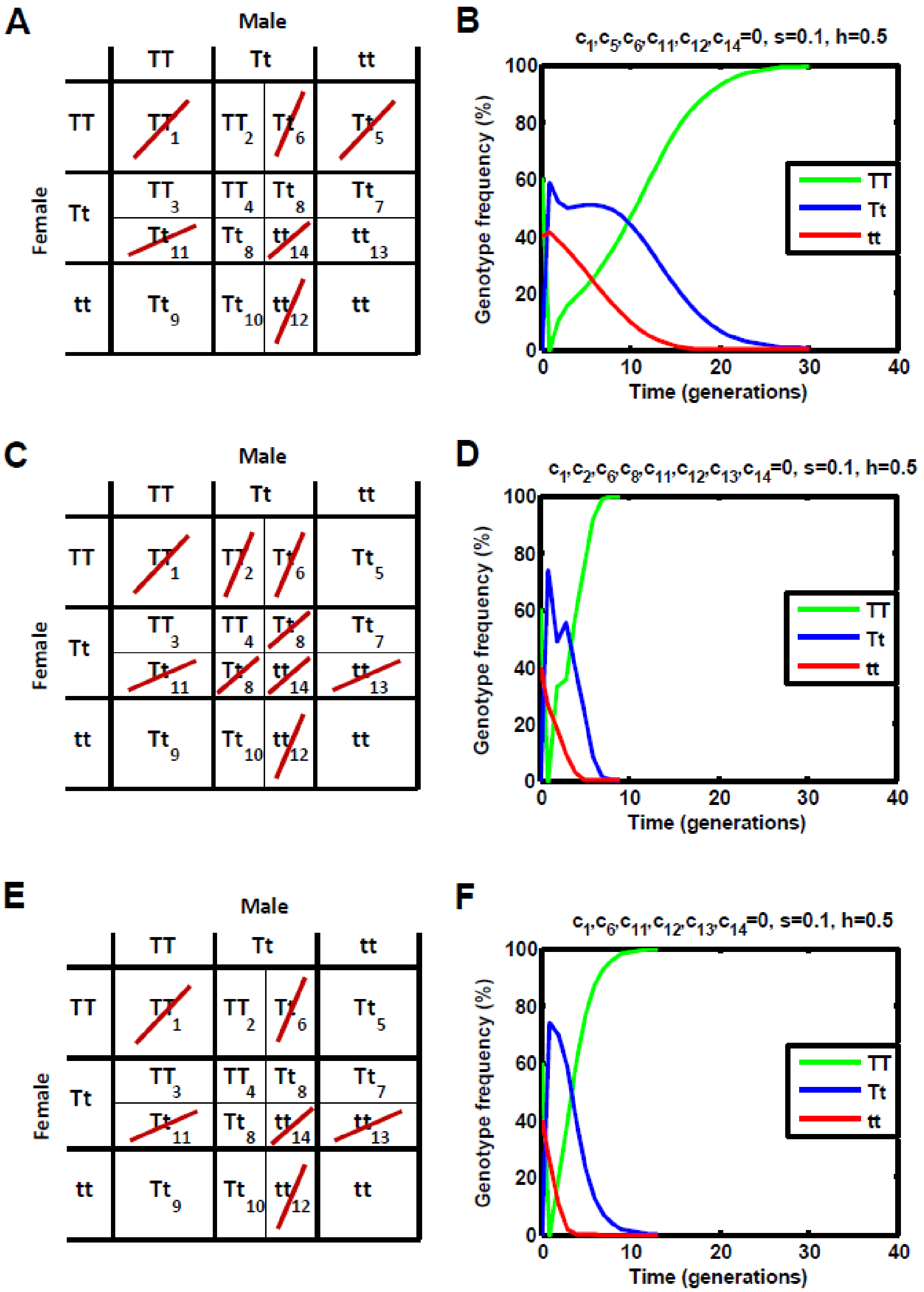

Figure S4. Examples of autosomal constructs for which crosses between *TT* males and *TT* females produce no viable offspring ($c_1 = 0$) that induce a population crash. A: The following construct provides one example ($c_1, c_5, c_6, c_{11}, c_{12}, c_{14} = 0$). B: Beginning from a

60% release, the population becomes entirely homozygous for the construct within 30 generations, resulting in a population crash because crosses between two *TT* parents produce no viable offspring. C: The following construct provides another example $(c_1, c_2, c_6, c_8, c_{11}, c_{12}, c_{13}, c_{14} = 0)$. D: Beginning from a 60% release, the population becomes entirely homozygous for the construct within 10 generations, leading to a population crash. E: The following construct provides a third example $(c_1, c_6, c_{11}, c_{12}, c_{13}, c_{14} = 0)$. F: Beginning from a 60% release, the population becomes entirely homozygous for the construct within 15 generations, leading to a population crash.

IV. X-linked constructs that lead to gene fixation or a population crash:

Figure S5:

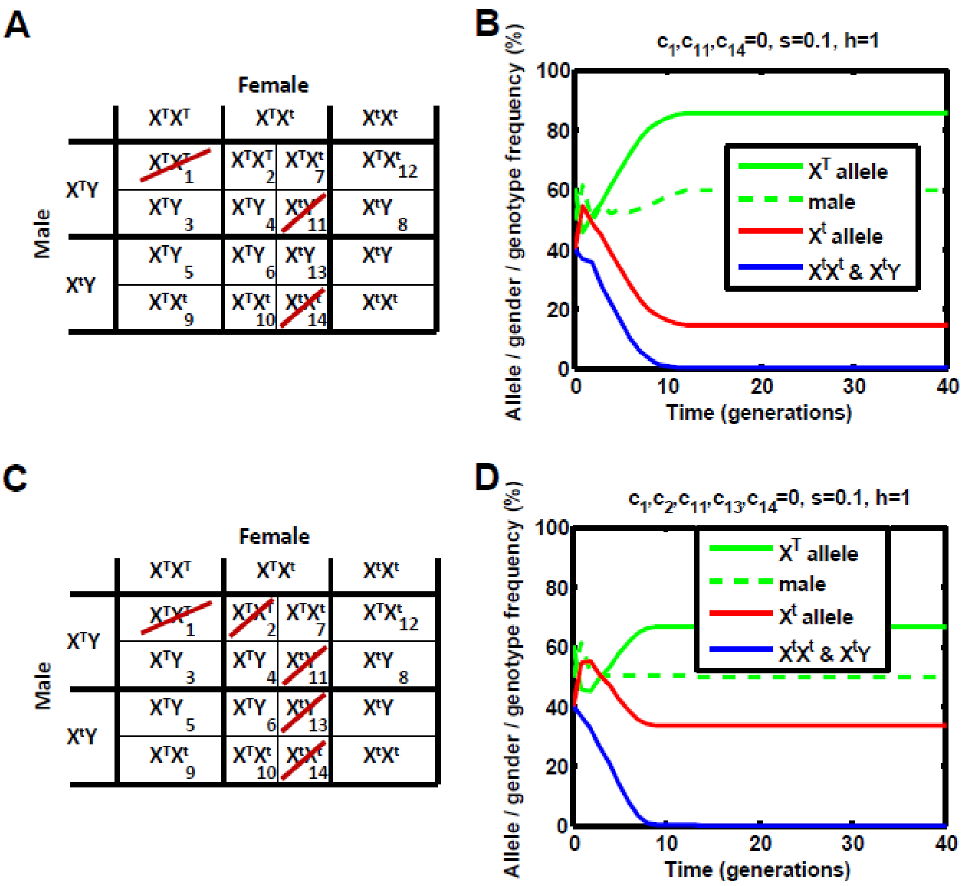

Figure S5. Examples of X-linked constructs for which crosses between X^TY males and X^TX^T females produce unviable male and/or female offspring ($c_1 = 0$ and/or $c_3 = 0$) that spread to gene fixation. A: The following construct provides one example ($c_1, c_{11}, c_{14} = 0$). B: Beginning from a 60% release, wild-type individuals are eliminated within 12 generations; however, the wild-type allele remains in the population. C: The following construct provides another example ($c_1, c_2, c_{11}, c_{13}, c_{14} = 0$) which could conceivably be engineered using a gene complex consisting of a dominant maternal toxin, a dominant zygotic toxin and a recessive zygotic antidote. D: Beginning from a 60% release, wild-type individuals are eliminated within 10 generations; however, the wild-type allele remains in the population.

Figure S6:

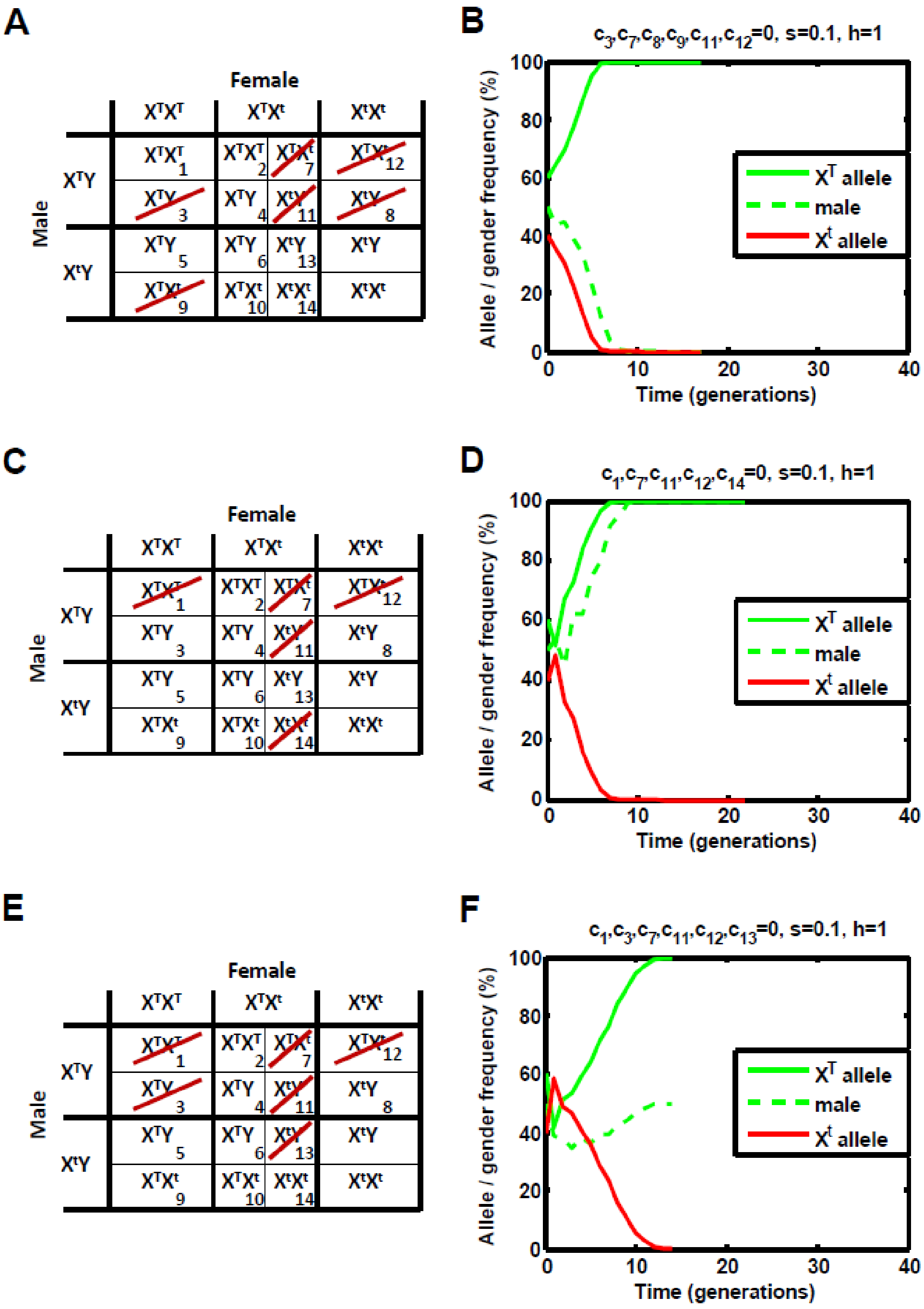

Figure S6. Examples of X-linked constructs for which crosses between X^TY males and X^TX^T females produce unviable male and/or female offspring ($c_1 = 0$ and/or $c_3 = 0$) that induce a population crash. A: The following construct provides an example of an all-

female population crash ($c_3, c_7, c_8, c_9, c_{11}, c_{12} = 0$). B: Beginning from a 60% release, the construct fixes and the population becomes entirely female within 10 generations, resulting in a population crash. C: The following construct provides an example of an all-male population crash ($c_1, c_7, c_{11}, c_{12}, c_{14} = 0$). D: Beginning from a 60% release, the construct fixes and the population becomes entirely male within 10 generations, resulting in a population crash. E: The following construct provides an example of a bi-sex population crash in which the population is driven towards male and female genotypes that produce no viable offspring ($c_1, c_3, c_7, c_{11}, c_{12}, c_{13} = 0$). F: Beginning from a 60% release, the construct fixes in the population within 15 generations, leading to a population crash.

V. X-linked toxin-antidote constructs:

Table S1:

X-linked toxin-antidote constructs that fix in a population despite a fitness cost

**Toxin:**	**Antidote:**	**Threshold:**
**The following X-linked constructs spread to allele fixation in the presence of modest fitness costs (results apply for general offspring lethality):**		
Dominant maternal toxin	Paternal antidote	55.8%
Dominant maternal toxin	Dominant zygotic antidote	0%
Recessive maternal toxin	Paternal antidote	66.7%
Dominant maternal toxin	Recessive maternal antidote	50%
Paternal toxin	Recessive maternal antidote	44.2%
Paternal toxin	Dominant zygotic antidote	0%
Dominant zygotic toxin	Recessive maternal antidote	66.7%
Dominant zygotic toxin	Paternal antidote	66.7%
**The following X-linked constructs spread to allele fixation in the presence of modest fitness costs (results apply for female-specific offspring lethality):**		
Dominant maternal toxin	Paternal antidote	36.1%
Dominant maternal toxin	Recessive zygotic antidote	40.0%
Recessive maternal toxin	Paternal antidote	50%
Recessive maternal toxin	Recessive zygotic antidote	50%
Dominant maternal toxin	Recessive maternal antidote	50%
Paternal toxin	Recessive maternal antidote	63.9%
Paternal toxin	Recessive zygotic antidote	50%
Dominant zygotic toxin	Recessive maternal antidote	66.7%
Dominant zygotic toxin	Paternal antidote	50%

Dominant zygotic toxin	Recessive zygotic antidote	50%
**The following X-linked constructs spread to allele fixation in the presence of modest fitness costs (results apply for male-specific offspring lethality):**		
Dominant maternal toxin	Dominant zygotic antidote	0%
Dominant maternal toxin	Recessive maternal antidote	50%
Paternal toxin	Recessive maternal antidote	3.8%
Paternal toxin	Dominant zygotic antidote	0%
**The following X-linked constructs induce an all-female population crash in the presence of modest fitness costs:**		
Paternal toxin (bi-sex-lethal)	Recessive zygotic antidote	50%
Paternal toxin (male-lethal) + Dominant maternal toxin (female-lethal)	Recessive zygotic antidote	40%
Paternal toxin (male-lethal) + Recessive maternal toxin (female-lethal)	Recessive zygotic antidote	50%
Paternal toxin (male-lethal) + Dominant zygotic toxin (female-lethal)	Recessive zygotic antidote	50%
Paternal toxin (male-lethal) + Dominant maternal toxin (male-lethal)	Recessive maternal antidote	50%
Paternal toxin (male-lethal) + Dominant maternal toxin (bi-sex-lethal)	Recessive maternal antidote	50%
Paternal toxin (male-lethal) + Dominant maternal toxin (female-lethal)	Recessive maternal antidote	50%

VI. References:

Elaydi, S. N. 1995. An Introduction to Difference Equations. Springer, New York.
